# Designing an intelligent push model for user emotional topics based on dynamic text categorization in social media news dissemination

**DOI:** 10.7717/peerj-cs.2607

**Published:** 2024-12-19

**Authors:** Jixuan Wang

**Affiliations:** School of Government and Public Affairs, Communication University of China, Beijing, China

**Keywords:** Social media, Text analysis, LSTM, Attention mechanism

## Abstract

The exploration of social media comment analysis has garnered considerable scholarly attention in recent epochs, precipitated by the pervasive ubiquity of social media platforms and the copious volume of commentaries engendered by their users. As the prevalence of users disseminating opinions, engaging in news discourse, and articulating sentiments on social media escalates, scrutinizing social media comments assumes paramount significance. This treatise employs a sophisticated deep network model for sentiment classification predicated on online social media textual commentary data, utilizing a bidirectional long short-term memory (BI-LSTM) network. The model initiates data input processing by employing word segmentation and word vector extraction, culminating in the formation of an attention-based bidirectional long short-term memory (ATT-Bi-LSTM) model, which incorporates an attention mechanism for discerning positive and negative emotions. Notably, the model attains recognition rates exceeding 80% for both categories of emotions within the public dataset. Concurrently, the model undergoes training migration for practical application validation using the public dataset, yielding recognition accuracy surpassing 90% in authentic testing scenarios. This substantiates the efficacy of the proposed methodology in proficiently accomplishing the emotion classification task within the dynamic text milieu of social media news propagation. Such proficiency, in turn, furnishes pivotal technical underpinnings for subsequent iterations of intelligent push models and astute public opinion analyses.

## Introduction

**Figure 1:**
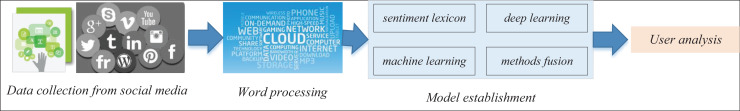
The user and public opinion analysis and supervision according to the social media.

As the expeditious and discerning comprehension of concise messages on social media platforms permeates public life, it progressively molds and rejuvenates the evolving landscape of online public opinion. In scrutinizing sentiment within this realm, prevailing machine learning methodologies of superior efficacy often hinge upon the annotation of substantial volumes of unprocessed textual data, subsequently extrapolating sentiment predictions onto test data. These training and test datasets typically emanate from akin domains, platforms, or contextual settings. However, these methodologies encounter heightened challenges in conducting sentiment analysis across disparate scenes or platforms and addressing opinion events that transcend domains. The apprehension of opinion dynamics in transposed settings assumes a heightened significance (Kalogeropoulos et al., 2017). Within social media journalism, a profound comprehension of users’ emotional responses to news events can provide media entities and decision-makers with invaluable insights into prevailing public attitudes and emotional orientations. Such discernment is pivotal for opinion surveillance, crisis management, and the recalibration of news communication strategies (Alrumaih et al., 2020). This inquiry’s essence lies in examining users’ pertinent discourses on social media, encapsulated as a subset of natural language processing predicaments. The comprehensive nature of this challenge is delineated in Fig. 1.

The establishment of word vectors and ancillary features is effectuated by employing crawlers and other technological modalities to amass textual information from online social media networks, accompanied by requisite processing. Subsequently, employing conventional lexicon models or contemporary methodologies such as machine learning and deep learning facilitates the categorization of tasks, culminating in comprehensive user analyses (Thelwall, 2018). Pertaining to sentiment recognition in the realm of deep learning, due consideration must be accorded to multimodal data, particularly encompassing textual, visual (image), and audio-visual (video) elements prevalent on social media platforms. Furthermore, the imperative of domain adaptation looms large, necessitating the model’s proficiency in adapting to the idiosyncrasies inherent in social media texts. Acknowledging the temporal dynamics intrinsic to dynamic textual content, the model is mandated to adeptly capture the nuanced evolution of sentiment over time. Mitigating the challenge of labeling imbalance assumes pivotal importance, and this is adroitly addressed through the judicious adoption of a fitting loss function strategy (Kastrati et al., 2021).

In the era of big data, research on sentiment classification of dynamic text has gone beyond the traditional domain of computer science, extending to management, social sciences, and other disciplines. Sentiment analysis of dynamic text has attracted significant attention in academic research and shows immense potential in commercial applications. Faced with vast, rapidly changing user review data on the internet, businesses need efficient and accurate sentiment classification to extract users’ emotional inclinations on platforms. Such analyses help identify user preferences, assess product and service quality, predict market trends, and ultimately provide users with more precise and high-quality services (Kastrati et al., 2021). Furthermore, this process provides crucial guidance for product innovation and optimization. Deep learning methods have become essential tools in sentiment classification tasks in this process due to their robust automatic feature-learning capabilities and end-to-end training. Models like recurrent neural networks (RNNs) and extended short-term memory networks (LSTMs) excel at capturing emotional fluctuations in review text over time, given their advantages in handling sequential data. While primarily used in image processing, convolutional neural networks (CNNs) have proven effective in sentiment analysis by capturing local features and emotional keywords within text.

Additionally, the recent application of pre-trained models, particularly bidirectional language models like BERT, has significantly improved the accuracy and efficacy of sentiment classification (Lin et al., 2021). Pre-trained on large-scale corpora, these models acquire rich semantic representations, enabling deeper emotional understanding in review sentiment classification. Supported by big data, these deep learning methods can efficiently process large-scale review data. However, these methods face several challenges as data volume and dimensionality increase. The first challenge lies in data cleaning and preprocessing. In dynamic text, user reviews often contain misspellings, abbreviations, emojis, and other non-standard text, complicating model processing. By combining extensive data methods with AI technologies, automated data-cleaning pipelines can extract high-quality, structured data from complex text, laying a solid foundation for subsequent sentiment classification. The diversity of data and the multidimensional nature of emotions also pose challenges for sentiment analysis. User reviews may express positive or negative emotions and mixed emotions, which requires sentiment classification models to have a solid contextual understanding. Attention mechanisms and multi-head self-attention in deep learning dynamically focus on key information within the text, enabling models to comprehend multiple meanings and emotional expressions.

Additionally, incorporating multimodal data (*e.g*., text, images, and audio) provides a more comprehensive perspective for sentiment classification, resulting in more accurate and detailed sentiment analysis outcomes. In the massive data environment, the computational demands of deep learning models also increase accordingly. The application of GPUs and TPUs has greatly accelerated model training, enabling businesses and researchers to obtain sentiment analysis results within an acceptable timeframe. Additionally, cloud-based distributed processing and large-scale data storage technology provide powerful computational and storage support for deep learning models, effectively meeting the high computational needs of big data analysis.

In summary, deep learning methods combined with big data analysis technologies provide an advanced solution for the sentiment classification of dynamic text. These technologies enhance classification accuracy and substantially reduce manual intervention costs, making large-scale sentiment analysis feasible in practical applications. Meanwhile, using techniques such as transfer learning and reinforcement learning can significantly expand the applicability of these models, thereby improving the effectiveness of sentiment classification. This study focuses on the analysis of review text in social media news dissemination, accomplishing sentiment classification of users, with the following specific contributions:

1) Proposing a sentiment classification model for social media comment data based on BI-LSTM, leveraging keyword vector information for enhanced model feature extraction and improved data performance.
2) Introducing the ATT-Bi-LSTM model, wherein weights are assigned to the BI-LSTM module through an attention mechanism. This augmentation facilitates the classification of positive and negative emotions within social media comment data.
3) Executing model training for migration learning in practical application scenarios, with experimental outcomes demonstrating the method’s adept utilization of existing datasets to achieve migration effects. The overall recognition accuracy surpasses 90%.

In the subsequent sections of this article, “Related works” delves into related work, while “Methodology” establishes the ATT-Bi-LSTM model. “Experiment Result and Analysis” comprehensively presents experimental results and associated analyses. Following that, “Discussion” constitutes the Discussion, with the conclusion drawn in the concluding section.

## Related works

### Text sentiment analysis based on sentiment lexicons and statistical method

Turney (2002) posit that the adjectives or adverbs within the commentary text exert the predominant influence on its overall semantic inclination. They gauge the semantic orientation of a word by computing the mean semantic orientation of its adjectives and adverbs. The specific methodology involves calculating the disparity in mutual information between a given adjective or adverb and the benchmark terms “excel” and “poor” to ascertain the word’s semantic orientation. Sista & Polarized (2004) similarly contend that the emotional polarity of words present in the commentary text shapes the overarching emotional polarity of the commentary. Upon amalgamating the General Inquiry and WordNet sentiment dictionaries, the sentiment classification of textual expressions related to products and services is grounded in the consolidated sentiment dictionaries. The outcomes indicate a noteworthy enhancement in text classification accuracy by using sentiment dictionaries. Kamps et al. (2004) assert that word distance or similarity measures derived from WordNet sentiment lexicons predominantly align with WordNet’s classification relationships, thereby confining their applicability within noun and verb syntactic categories. Pang, Lee & Vaithyanathan (2002), utilizing extensive film reviews as a *corpus*, employed three machine learning algorithms—namely, support vector machine (SVM), naive Bayes (NB), and maximum entropy (ME)—for sentiment classification. The findings reveal that the SVM algorithm exhibits superior sentiment classification performance; nevertheless, all three algorithms underperform compared to topic-based classification methodologies. Ye, Zhang & Law (2009), leveraging blog comments within the tourism domain as their *corpus*, juxtaposed sentiment classification performance using three machine learning algorithms: SVM, NB, and a character-based N-gram model. The results demonstrate that naive Bayes manifests the least satisfactory classification performance among the trio.

Nevertheless, with a sufficiently extensive training set sample, the accuracy of these methods could surpass 80%. Mukwazvure & Supreethi (2015) adopted a hybrid approach for sentiment classification of news comments. Initially, sentiment tendencies were categorized into positive, negative, and neutral. Subsequently, the classification outcomes based on sentiment dictionaries served as training data for machine learning algorithms such as SVM and k-neural network (KNN). The findings highlight the superiority of the SVM algorithm over the KNN algorithm in the sentiment classification of news comments. Rohini, Thomas & Latha (2016) synthesized sentiment lexicons and decision tree classifiers to execute sentiment classification on a dataset of movie reviews. They compared sentiment classification results using the Kannada language movie review dataset and the machine-translated English review dataset.

Nevertheless, conventional methodologies for analyzing textual sentiment, relying on established sentiment dictionaries, manifest conspicuous deficiencies. Such strategies hinge on a restricted lexicon, posing a formidable challenge in encompassing diverse contexts and burgeoning lexicons. This limitation hinders capturing emotional nuances in specific domains or contemporary occurrences. Secondly, traditional approaches grapple with the inherent ambiguity of words, as a term may evoke varied emotions in disparate contexts. Resolving such ambiguity proves formidable, detrimentally impacting the precision of sentiment analysis.

Furthermore, conventional methods frequently disregard the contextual influence on emotion, neglecting to consider the amalgamation of adjacent words and syntactic structures adeptly. This oversight constrains a profound comprehension of the sentiment embedded within the text. Moreover, the emotional polarity inherent in sentiment dictionaries often stems from subjective judgments, introducing bias that impedes adaptability to diverse manifestations of emotion across varied demographics or cultures. Consequently, with the progression of deep learning technologies, textual content analysis can undergo marked improvement through protracted scrutiny of time-series data.

### Text sentiment analysis based on deep learning methods

McDonald et al. (2007) conducted an inquiry into a structured model for text sentiment classification that operates at different levels of granularity collaboratively. The distinctive advantage of this model lies in its ability to correlate and mutually influence classification results across various text levels. Empirical experiments have demonstrated a notable reduction in classification errors compared to isolated training models (McDonald et al., 2007). Fei, Liu & Wu (2004) introduced a classification method that combines phrase patterns with unsupervised learning algorithms to analyze the sentiment tendencies in sports comment data on specific websites. The results substantiate the method’s efficacy, showcasing commendable classification outcomes (Fei, Liu & Wu, 2004). Kennedy & Inkpen (2006) leveraging semantic and machine learning algorithms, proposed a sentiment classification method for online movie review texts. The findings underscore that the amalgamation of these two methodologies yields superior results compared to their individual use (Kennedy & Inkpen, 2006). Thet, Na & Khoo (2008) advocated combining information extraction techniques and machine learning in sentiment classification for in-depth analysis of movie review texts. Experimental results affirm the effectiveness of this method in the sentiment classification of online movie reviews (Thet, Na & Khoo, 2008). Hajmohammadi et al. (2015) introduced an innovative learning model that combines uncertainty-based active learning and semi-supervised self-training methods to classify sentiment in online book review data across three different languages.

The outcomes demonstrate a significant enhancement in the effectiveness of cross-lingual sentiment classification (Hajmohammadi et al., 2015). In recent years, the attention mechanism, recognized as an efficient technique, has seamlessly transitioned from image recognition to natural language processing. Its integration with deep learning techniques has proven to enhance the effectiveness of sentiment classification models. Usama et al. (2020) proposed a novel model based on RNN and CNN, incorporating the attention mechanism. Basiri et al. (2021) introduced an attention-based bi-directional CNN-RNN model that assigns weights to the bi-directional feature extraction layer using the attention mechanism. Liu et al. (2021) incorporated positional coding alongside a multi-head attention mechanism, employing graph convolutional networks to capture sentiment information from text, address interactions between contextual and aspectual words, and analyze long-distance dependencies between words. Zhu et al. (2021) introduced global and local textual information simultaneously into aspect-based sentiment classification, utilizing the attention mechanism to merge global and local features effectively.

The research above underscores that owing to the exponential surge in network information, traditional model-based approaches to social media opinion and sentiment analysis are increasingly inadequate for the requirements of pertinent departments. Consequently, the evolution of artificial intelligence-based methods, rooted in machine learning and deep learning, has emerged as a pivotal solution to address these challenges. Following data acquisition, the obtained data undergoes analysis using deep learning models for sentiment analysis. Enhancing the model’s efficacy through data reinforcement, achieved *via* reinforcement learning and other methodologies, markedly improves data performance and the model’s recognition capabilities. This progression offers valuable technical insights for the nuanced sentiment analysis of dynamic text.

## Methodology

### The BI-LSTM for the text recognition

For sentiment analysis on textual data, the crux lies in addressing a time series classification problem, making RNNs the optimal solution within deep learning. To better facilitate the study of contextual semantics, this article employs a BI-LSTM network for model construction. LSTM is a specialized variant of recurrent neural networks expressly designed to overcome challenges associated with long sequence dependencies (Shi et al., 2022). By incorporating a gating mechanism, LSTM adeptly captures and manages prolonged dependencies in sequences, mitigating issues such as gradient vanishing or explosion that afflict traditional RNNs when handling extensive sequences.

The LSTM unit predominantly executes data optimization through an oblivious gate unit, ensuring effective long-term control. The implementation process of the oblivious gate is delineated in Eq. (1):

(1) $${f_t} = \sigma \left( {{W_f} \cdot \left[ {{h_{t - 1}},{x_t}} \right] + {b_f}} \right)$$

where ${W_f}$ is the weight matrix. ${h_{t - 1}}$ is the hidden state. ${x_t}$ is the input of at current time. The computation of the hidden state involves the output from the preceding stage and the corresponding memory cells, as depicted by Eq. (2):

(2) $${h_t} = {o_t} \cdot {\rm tanh}\left( {{C_t}} \right)$$

where *C_t_* is the state of the cell at time step t. It is related to the computation of the forgetting gate, which can be calculated by Eq. (3):

(3) $${C_t} = {f_t} \cdot {C_{t - 1}} + {i_t} \cdot \tanh \left( {{W_c} \cdot \left[ {{h_{t - 1}},{x_t}} \right] + {b_c}} \right).$$

Upon completing the aforementioned calculations for the pertinent state variables, the output of each cell, and consequently the final output, can be obtained:

(4) $${o_t} = \sigma \left( {{W_o} \cdot \left[ {{h_{t - 1}},{x_t}} \right] + {b_o}} \right).$$

**Figure 2:**
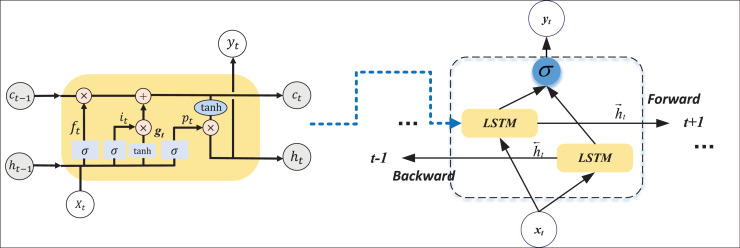
The structure for the BI-LSTM.

BI-LSTM represents an extension of LSTM, enhancing the capture of contextual information by assimilating both forward and reverse information from the input sequence. The hidden states of BI-LSTM are concatenated, incorporating information from both forward LSTM and reverse LSTM. By processing information in both left-to-right and right-to-left directions, BI-LSTM facilitates a more comprehensive understanding of the entire sequence. This bidirectional approach enables the model not only to consider past information but also to anticipate future information. The computational process of BI-LSTM is elucidated in Eqs. (5), (6):

(5) $$\overrightarrow {{h_t}} = \overrightarrow {{\rm LSTM}} \left( {{x_t},{h_{t - 1}}} \right) = \sigma \left( {W \cdot \left[ {{h_{t - 1}},{x_t}} \right] + b} \right)$$

(6) $$\overleftarrow {{h_t}} = \overleftarrow {{\rm LSTM}} \left( {{x_t},\overleftarrow {{h_{t + 1}}} } \right) = \sigma \left( {{\rm W} \cdot \left[ {\overleftarrow {{h_{t + 1}}} ,{x_t}} \right] + {\rm b}} \right)$$

where ${h_t}$ is the time step $t$ of the hidden state, and $\overrightarrow {{h_t}}$ is the output of forward and $\overleftarrow {{h_t}}$ is the output of the backward, and the process of merging the two outputs in the sequence is shown in the right part of Fig. 2.

BI-LSTM exhibits significant advantages in sequence modeling tasks. Simultaneously processing forward and backward information, BI-LSTM excels at capturing contextual relationships more comprehensively, providing richer contextual information. This attribute is particularly advantageous for tasks involving long-distance dependencies. The bidirectional structure enhances the model’s ability to learn abstract representations of sequences, mitigates the rate of information loss, maintains symmetry balance, and elevates the stability and robustness of the model. BI-LSTM finds practical application in natural language tasks for recognizing patterns and analyzing data in context. Its capacity to consider contextual information on both the left and right sides of a word simultaneously enables a more comprehensive understanding of context and the capture of long-distance dependencies in text. Given the uncertainty and variability in text length, BI-LSTM exhibits flexibility in adapting to indeterminately long input sequences. It demonstrates the ability to learn features, rendering it well-suited for analyzing input and output in related data.

### The attention mechanism for the model enhancement

**Figure 3:**
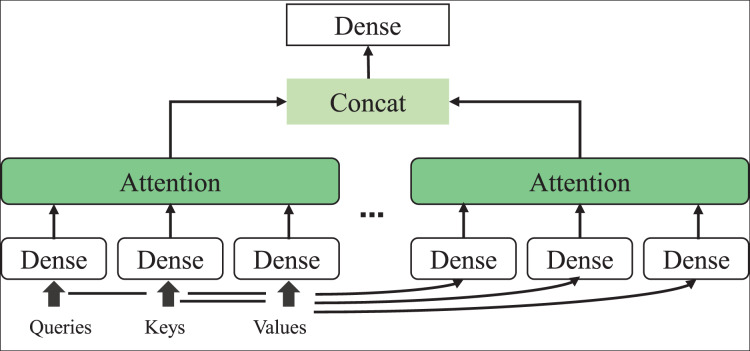
The schematic diagram of attention principle.

The attention mechanism emulates how the human brain filters critical information and constructs probability distribution matrices to associate features that require learning. Initially proposed for application in image processing, the attention mechanism has since found widespread use in natural language processing tasks. Operating as a weight distribution mechanism, the attention mechanism can extract critical information from extensive data and adjust the significance of text features by modifying the weight coefficients (Niu, Zhong & Yu, 2021). The larger the weight coefficient, the more crucial the information is deemed in the comment text, exerting a more significant impact on the sentiment polarity classification results of the text. Assuming Q is a matrix composed of a set of queries, K is a matrix consisting of a set of keys, and V is a matrix composed of a set of values, the attention mechanism, denoted as Attention (Q, K, V), is delineated in Eq. (7):

(7) $${\rm Attention}(Q,K,V)=\sum\limits_{i=1}^{m}{{1}\over{z}}{\rm exp}\left({{< q_{t},k_{i}\gt}\over{\surd}d_k}\right)v_{i}$$

where $Z$ is the normalization factor, and ${d_k}$ is the dimension of the word embedding vector, which can play the role of adjusting factor in the formula so that the inner product will not be too large. ${q_t}$ (query) is the task to be queried, and ${k_i}$ (key) and ${v_i}$ (value) are the corresponding key-value pairs. ${q_t}$ by calculating the inner product. The entire process can be visually represented in Fig. 3. These scores are normalized into attention weights using the softmax function for different key values Q, K, and V. These weights are then utilized to weigh and sum the values in the input sequence, ultimately generating the final attention output. This dynamic attention mechanism enables the model to selectively focus on information at various locations within the input sequence, enhancing its capability to capture key information. In this article, incorporating the attention mechanism involves augmenting the feature vocabulary based on the results obtained from the BI-LSTM method, thereby completing the enhanced training of the model.

In sentiment analysis of social media content, the introduction of attention mechanisms is precious due to user-generated text’s diverse and often unstructured nature. Social media posts vary widely in length, structure, and emotional complexity, with important sentiment cues sometimes embedded within specific phrases or keywords. Attention mechanisms enable the model to dynamically focus on these relevant portions of text, significantly enhancing its ability to capture critical features and contextual information essential for accurate sentiment interpretation. Furthermore, attention mechanisms improve the model’s adaptability to the informal language and mixed sentiments commonly found in social media, leading to more robust sentiment classification. This dynamic focus also contributes to the model’s interpretability, allowing researchers and practitioners to gain insight into which words or phrases most influence the model’s decision-making process, strengthening confidence in the analytical results.

### The ATT-BI-LSTM for the social media text recognition

**Figure 4:**
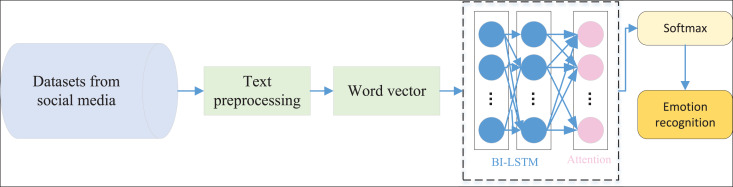
The framework for the social media text analysis.

After completing the introduction of the BI-LSTM model and the attention mechanism, we carried out the construction of the model, and the overall model framework is shown in Fig. 4.

The model first processes social media-related data collected from public datasets, involving data cleaning, keyword analysis, and text vectorization to ensure the data is high quality and structured appropriately for model input. During data cleaning, redundant characters, stopwords, punctuation, and irrelevant information are removed to reduce noise and maintain data purity. For keyword analysis, techniques like word frequency statistics and TF-IDF are used to extract core terms from each text, allowing for a more accurate reflection of the text’s themes and sentiments. To efficiently represent text semantics, we vectorize the text data using pre-trained word embedding models such as Word2Vec or GloVe, ensuring each word is mapped into a multidimensional vector space, preserving the semantic and contextual information of the text. The dimensionality of the word vectors is specifically set to capture emotional features, balancing generalization ability and ease of data processing. The processed word vectors are then fed into the model, with the input layer’s maximum text length set to 512 to capture the complete information in most comments. The model uses a two-layer BI-LSTM structure, each containing 128 LSTM units, designed to capture contextual sentiment information in both forward and backward directions. Additionally, an attention mechanism is incorporated, allowing the model to adaptively focus on essential words and phrases, thereby enhancing the capture of emotional features and improving sentiment classification accuracy. In the classification stage, Softmax and a fully connected layer are used to perform binary classification of sentiment, identifying positive or negative emotions within the text.

## Experiment result and analysis

### Experiment setup

To enhance the model’s validation and training, a public dataset was utilized for analysis and pre-training under migration learning, facilitating the testing of the model on real-world data. The microblogging dataset employed in this study is the GitHub public microblogging sentiment analysis dataset (https://github.com/SophonPlus/ChineseNlpCorpus/blob/master/datasets/weibo_senti_100k/intro.ipynb.), containing sentiment annotations for Sina microblogs, totaling over 100,000 items. The dataset exhibits an equal distribution of negative and positive sentiments, each accounting for half of the entries. For practical application considerations, data entries were filtered, and 2,000 positive comments and 2,000 negative comments were selected for analysis.

In addition, the IMDB review dataset (Tripathi et al., 2020) was employed, representing a binary categorical sentiment dataset with 50,000 IMDB film reviews. Reviews with a negative score of <= 4 and a positive score of >= 7 were included. Given the volume of data, 2,000 positive and 2,000 negative reviews were chosen for analysis, thereby completing the model evaluation.

Following the dataset determination, corresponding indicators for model evaluation were selected. Precision, Recall, and F1 score were chosen as the evaluation metrics in this article. The calculation methods for these indicators are delineated in Eqs. (8)–(10):

(8) $$P = \displaystyle{{TP} \over {TP + FP}}$$

(9) $$R = \displaystyle{{TP} \over {TP + FN}}$$

(10) $$F1 = \displaystyle{{2 \times P \times R} \over {P + R}}$$

where $TP$ denotes a true example; $FP$ denotes a false positive example; and $FN$ denotes a false negative example. In this model, careful tuning of hyperparameters was essential to achieve optimal performance in sentiment classification. The input layer was set with a maximum text length of 512, balancing the need to capture context with computational efficiency. Each of the two layers in the BI-LSTM model was configured with 128 LSTM units, a choice aimed at maintaining a balance between capturing sufficient sequential information and managing model complexity to avoid overfitting. The attention mechanism enhanced the model’s focus on crucial features, improving sentiment detection. The batch size for training was set to 10, allowing efficient updates while maintaining stability in gradient descent. The Adam optimizer was chosen for its adaptability to varying learning rates, enhancing convergence speed. The classification was finalized with a Softmax layer, clearly distinguishing between positive and negative sentiments. This combination of hyperparameters helped the model achieve robust performance while efficiently processing the social media data for accurate sentiment classification.

### Emotion classification result and model comparison

**Figure 5:**
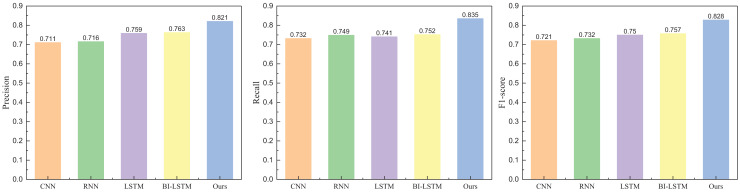
The positive emotion recognition result on Weibo dataset among different methods.

**Figure 6:**
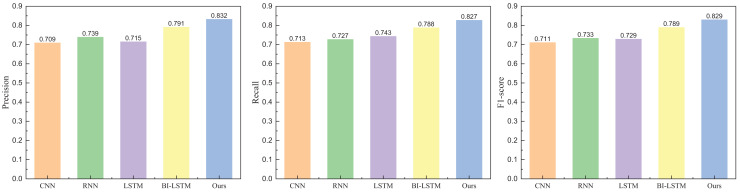
The negative emotion recognition result on Weibo dataset among different methods.

Following the completion of the model’s dataset and evaluation indicators, we proceeded with the model testing. In the comparative analysis of models, we selected commonly used methods in natural language processing, including CNN, RNN, LSTM, and BI-LSTM. The model-building parameters, the number of layers, and the proposed model have been aligned. The specific results obtained from Weibo data are illustrated in Figs. 5 and 6.

**Figure 7:**
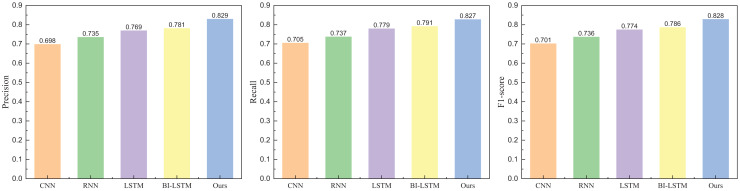
The positive emotion recognition result on IMDB dataset among different methods.

**Figure 8:**
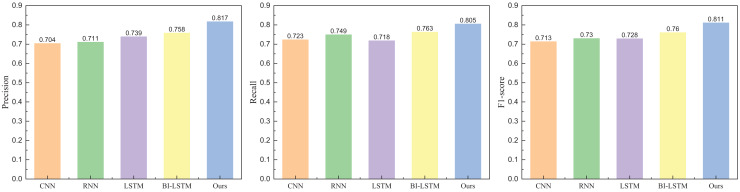
The negative emotion recognition result on IMDB dataset among different methods.

In Figs. 5 and 6, it is evident that the method proposed attains elevated recognition results in both positive and negative emotion recognition. The precision is 0.821 for positive emotion recognition and 0.832 for negative emotion recognition, demonstrating commendable recognition outcomes. Conversely, without adding the attention module, the method exhibits a diminished recognition rate. The recognition results for another dataset, IMDB, are depicted in Figs. 7 and 8.

**Figure 9:**
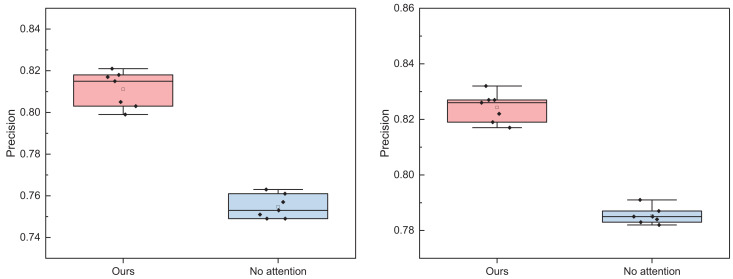
The ablation experiment for the positive and negative emotion on Weibo datasets.

**Figure 10:**
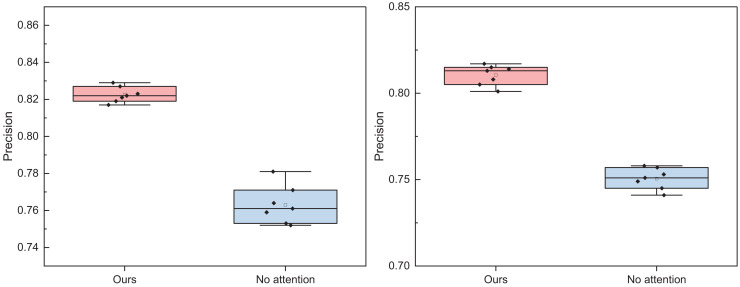
The ablation experiment for the positive and negative emotion on IMDB datasets.

In Figs. 7 and 8, the addition of the attention module contributes to an overall improvement in recognition rates. After incorporating the attention module, the precision for positive and negative emotions is 0.829 and 0.817, respectively. Both metrics demonstrate significant enhancements due to the individual contributions of the attention module. In contrast, the precision of BI-LSTM without the attention module is 0.781 and 0.758, indicating a substantial improvement in the model’s overall performance after incorporating the relevant module. To further validate the impact of the attention mechanism module on model performance, ablation experiments were conducted, and the results are illustrated in Figs. 9 and 10.

In Figs. 9 and 10, it is evident that whether on the Weibo dataset or the IMDB dataset, the addition of the attention module consistently yields superior overall recognition results across different batches compared to the standalone BI-LSTM model. The recognition accuracy is more consistent, resulting in an overall improved performance. Hence, the multi-faceted model proposed proves to be more adept at accomplishing the task of emotion recognition in dynamic text comments on social media news, thereby enhancing opinion analysis.

### The practical test for the model

**Figure 11:**
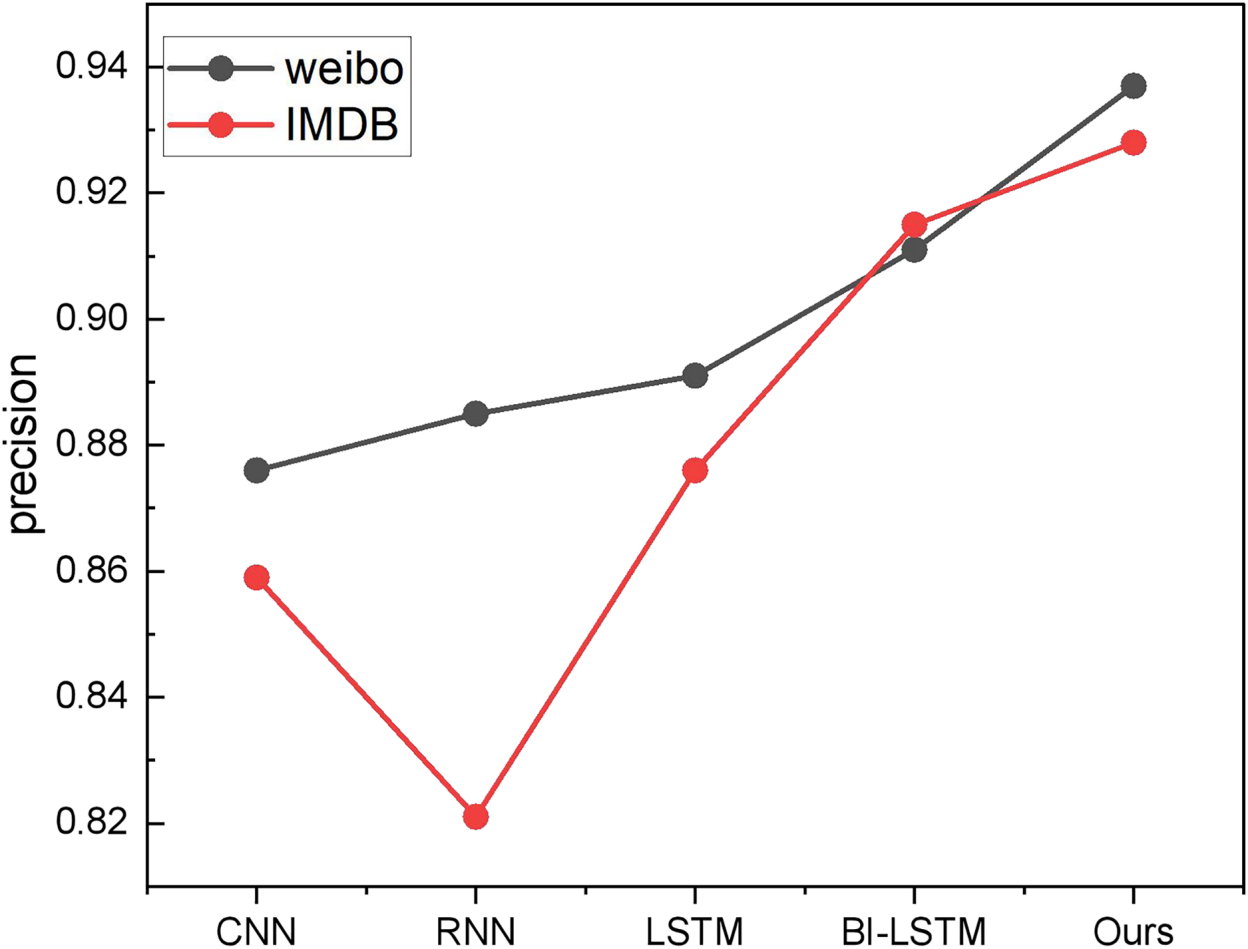
The transfer learning using Weibo and IMDB for the practical test.

As per this article’s requirements, we analyzed the APP data of interest sourced from news comments over the past 3 years. Approximately 1,000 entries were selected for data analysis, and the labels for these data entries were manually assigned. Simultaneously, to augment the dataset, we utilized Weibo data and IMDB data for separate training and observation through transfer learning. The recognition rates of different models obtained after transfer learning are presented in Table 1 and Fig. 11.

**Table 1:** The transfer learning using Weibo and IMDB for the practical test.

Datasets	CNN	RNN	LSTM	BI-LSTM	Ours
Weibo	0.876	0.885	0.891	0.911	0.937
IMDB	0.859	0.821	0.876	0.915	0.928

From the curves shown in the figure, we can observe that the transfer effect obtained after pre-training the model on the same data is similar, indicating that the proposed ATT-Bi-LSTM method achieved favorable recognition performance. The average precision for both positive and negative classifications exceeded 90%, demonstrating the effectiveness of this method in the required application scenarios of this study. We constructed a Python-based deep learning environment for model deployment, primarily using TensorFlow and PyTorch frameworks, running on high-performance servers to ensure efficient model training and testing. The data preprocessing pipeline includes tokenization, stopword removal, and lemmatization, leveraging libraries like NLTK and SpaCy for accurate text processing. For word vector initialization, we used pre-trained embeddings such as GloVe and Word2Vec to capture deep semantic relationships within the text, thereby improving the model’s initial performance. To ensure consistency and ease of deployment across various environments, the model was encapsulated in a Docker container, enabling rapid deployment in development, testing, and production environments. Kubernetes was employed for container orchestration, allowing for automatic scaling as needed. Data processing and model training operations were conducted within the container to maintain a consistent environment, reducing dependency issues.

Additionally, model performance evaluation was conducted using cross-validation, with continuous hyperparameter tuning to enhance model accuracy. For transfer learning, we initially trained the pre-trained model on source domain data, fine-tuning the target domain and leveraging Weibo and IMDB datasets to enhance cross-domain sentiment classification. This deployment environment enabled efficient data processing and model updating, establishing a robust foundation for future large-scale applications.

## Discussion

In this study, we address the challenge of sentiment recognition in dynamic text, specifically comment data, within social media news dissemination. Our research and analysis of current machine learning methods with superior performance reveal that many rely on annotating large volumes of raw text data for sentiment prediction and analysis. Typically, both the training and test sets are derived from the same domain, platform, or scenario data, making it challenging for these methods to conduct sentiment analysis in scenarios involving migration or cross-platform and cross-domain public opinion events (Anstead & O’Loughlin, 2015). The ATT-Bi-LSTM social media sentiment intelligent analysis model applied in this article demonstrates the ability to accurately capture long-distance dependencies and semantic features, addressing the challenges associated with text data labeling to a certain extent. This model facilitates sentiment analysis research across different scenarios through cross-scene migration learning.

Additionally, we conduct thorough comparisons with fundamental deep learning models such as CNN, LSTM, and RNN to ensure the validity of experimental results and enhance the accuracy and credibility of the overall findings. Compared to traditional machine learning methods based on dictionaries and word vectors, deep learning methods for word segmentation achieve higher accuracy in sentiment recognition. The migration model experiments validate that superior recognition results can be attained through straightforward pre-training. The misclassification observed in this model appears to be mainly due to challenges in capturing nuanced context and shifts in sentiment within social media texts, especially in cases involving sarcasm or mixed emotions. Furthermore, the model encounters difficulty with informal language, slang, and abbreviations frequently used in social media, which can obscure clear sentiment indicators and create ambiguity. These factors highlight areas where the model’s interpretative capacity may be limited, impacting overall accuracy in sentiment classification.

In future research on dynamic text in social media, key considerations should be given to factors such as data quality, appropriate text preprocessing, clear sentiment label definitions, and careful model selection. It is imperative to ensure that the model can handle domain specificity and that its performance is regularly assessed using appropriate evaluation metrics. Future research directions include implementing transfer learning on the model, exploring multimodal sentiment analysis, and incorporating long-term dependency modeling to enhance adaptability and comprehensiveness (Liu et al., 2020). Anticipated technological advancements include improvements in multimodal capabilities, adaptability, personalization, and interpretability of models to meet the demands of complex and evolving real-world applications (Salleh, 2017). Breakthroughs are expected in multimodal sentiment analysis, personalized sentiment recognition, and long-text processing. Simultaneously, research focusing on model transparency, prevention of adversarial attacks, and context-awareness is essential to fortify sentiment recognition technology, making it more robust and adaptable to diverse application scenarios. Future developments also involve enhancing real-time and dynamic processing, personalized push strategies, social network relationship mining, semantic understanding and reasoning, and addressing ethical and privacy considerations and cross-cultural and cross-linguistic adaptations (Li & Xu, 2020). Comprehensive and accurate sentiment analysis algorithms, integrating multiple information sources for personalized tweets, and considering users’ emotional expressions and topic concerns in different social contexts will be pivotal. Ethical and privacy issues must be carefully considered to respect users’ rights and interests, ensuring the sustainable development of intelligent social media communication systems.

## Conclusion

As a rapidly evolving research field, social media comment analysis has witnessed significant advancements fueled by technologies such as deep learning and natural language processing. A more comprehensive understanding of users’ perspectives, sentiments, and attitudes is achievable through a systematic examination of social media comments. This understanding provides insights into social opinions, aids in trend prediction, and offers robust information support for decision-makers. This article establishes an ATT-Bi-LSTM network model for sentiment classification of comments in social media-based news content dissemination. The model utilizes comment word vectors as inputs, employs BI-LSTM with two-layer units for classification, and enhances category weights using the ATTENTION module. This approach completes the classification of POSITIVE and NEGATIVE emotions. In testing on a public dataset, the proposed model achieves an average recognition rate of 0.827 and 0.823 for positive and negative emotions, respectively, surpassing traditional methods in overall recognition rate. In practical tests involving model pre-training through migration learning, the precision exceeds 90%, outperforming other techniques with a more stable recognition effect. This presents a novel reference for future social network media text analysis.

In cross-domain sentiment classification, the scarcity of high-quality labeled datasets and the prevalence of large amounts of unlabeled data significantly impact the effectiveness of text sentiment classification. Future research should focus on developing more generalizable pre-trained models that can adapt to diverse domains without requiring extensive domain-specific adjustments. This involves exploring advanced techniques such as domain adaptation, transfer learning, and self-supervised learning to maximize the usability of unlabeled data and reduce dependency on annotated datasets. Additionally, designing more efficient feature extraction structures is crucial for enhancing cross-domain applicability, as traditional models often struggle to capture the nuanced, domain-specific features necessary for accurate sentiment detection.

Another promising direction is the integration of multimodal data sources, such as combining text with images, audio, or contextual metadata, which may provide complementary information and enrich the feature space for sentiment classification tasks. Furthermore, researching improved fine-tuning techniques for pre-trained models can enable better handling of the variability in language, sentiment expressions, and cultural differences across domains. Advances in these areas could lead to more robust sentiment classification models adaptable to various industries, from social media and e-commerce to healthcare and finance, thereby widening the scope and impact of cross-domain sentiment analysis applications.

## Supplemental Information

10.7717/peerj-cs.2607/supp-1 Supplemental Information 1 Code.

## Additional Information and Declarations

### Data Availability

The following information was supplied regarding data availability: The code is available in the Supplemental File. The IMDB Reviews are available at Zenodo: N/A. (2021). IMDB Reviews (Version v1) [Data set]. Zenodo. https://doi.org/10.5281/zenodo.7555547. The dataset for summarization of microblogs is available at Zenodo: Soumi Dutta, Vibhash Chandra, Kanav Mehra, Asit Kumar Das, Tanmoy Chakraborty, & Saptarshi Ghosh. (2018). Dataset for summarization of microblogs [Data set]. In IEEE Intelligent Systems (Vol. 33, Number 3, pp. 4–14). Zenodo. https://doi.org/10.5281/zenodo.4430109.
