# Supplementary material for: Designing an intelligent push model for user emotional topics based on dynamic text categorization in social media news dissemination
Source: PeerJ Comput Sci. 2024 Dec 19;10:e2607. doi: 10.7717/peerj-cs.2607 (PMC11784813; doi:10.7717/peerj-cs.2607)
Supplement: Supplemental Information 1 [file peerj-cs-10-2607-s001.zip › code/attModel1/1409.0473.pdf]

NEURAL MACHINE TRANSLATION BY JOINTLY LEARNING TO ALIGN AND TRANSLATE

Dzmitry Bahdanau
Jacobs University Bremen, Germany

KyungHyun Cho **Yoshua Bengio***
Université de Montréal

arXiv:1409.0473v7 [cs.CL] 19 May 2016

ABSTRACT

Neural machine translation is a recently proposed approach to machine translation. Unlike the traditional statistical machine translation, the neural machine translation aims at building a single neural network that can be jointly tuned to maximize the translation performance. The models proposed recently for neural machine translation often belong to a family of encoder–decoders and encode a source sentence into a fixed-length vector from which a decoder generates a translation. In this paper, we conjecture that the use of a fixed-length vector is a bottleneck in improving the performance of this basic encoder–decoder architecture, and propose to extend this by allowing a model to automatically (soft-)search for parts of a source sentence that are relevant to predicting a target word, without having to form these parts as a hard segment explicitly. With this new approach, we achieve a translation performance comparable to the existing state-of-the-art phrase-based system on the task of English-to-French translation. Furthermore, qualitative analysis reveals that the (soft-)alignments found by the model agree well with our intuition.

1 INTRODUCTION

Neural machine translation is a newly emerging approach to machine translation, recently proposed by Kalchbrenner and Blunsom (2013), Sutskever *et al.* (2014) and Cho *et al.* (2014b). Unlike the traditional phrase-based translation system (see, e.g., Koehn *et al.*, 2003) which consists of many small sub-components that are tuned separately, neural machine translation attempts to build and train a single, large neural network that reads a sentence and outputs a correct translation.

Most of the proposed neural machine translation models belong to a family of *encoder–decoders* (Sutskever *et al.*, 2014; Cho *et al.*, 2014a), with an encoder and a decoder for each language, or involve a language-specific encoder applied to each sentence whose outputs are then compared (Hermann and Blunsom, 2014). An encoder neural network reads and encodes a source sentence into a fixed-length vector. A decoder then outputs a translation from the encoded vector. The whole encoder–decoder system, which consists of the encoder and the decoder for a language pair, is jointly trained to maximize the probability of a correct translation given a source sentence.

A potential issue with this encoder–decoder approach is that a neural network needs to be able to compress all the necessary information of a source sentence into a fixed-length vector. This may make it difficult for the neural network to cope with long sentences, especially those that are longer than the sentences in the training corpus. Cho *et al.* (2014b) showed that indeed the performance of a basic encoder–decoder deteriorates rapidly as the length of an input sentence increases.

In order to address this issue, we introduce an extension to the encoder–decoder model which learns to align and translate jointly. Each time the proposed model generates a word in a translation, it (soft-)searches for a set of positions in a source sentence where the most relevant information is concentrated. The model then predicts a target word based on the context vectors associated with these source positions and all the previous generated target words.

*CIFAR Senior Fellow

The most important distinguishing feature of this approach from the basic encoder–decoder is that it does not attempt to encode a whole input sentence into a single fixed-length vector. Instead, it encodes the input sentence into a sequence of vectors and chooses a subset of these vectors adaptively while decoding the translation. This frees a neural translation model from having to squash all the information of a source sentence, regardless of its length, into a fixed-length vector. We show this allows a model to cope better with long sentences.

In this paper, we show that the proposed approach of jointly learning to align and translate achieves significantly improved translation performance over the basic encoder–decoder approach. The improvement is more apparent with longer sentences, but can be observed with sentences of any length. On the task of English-to-French translation, the proposed approach achieves, with a single model, a translation performance comparable, or close, to the conventional phrase-based system. Furthermore, qualitative analysis reveals that the proposed model finds a linguistically plausible (soft-)alignment between a source sentence and the corresponding target sentence.

2 Background: Neural Machine Translation

From a probabilistic perspective, translation is equivalent to finding a target sentence $\mathbf{y}$ that maximizes the conditional probability of $\mathbf{y}$ given a source sentence $\mathbf{x}$, i.e., $\arg\max_{\mathbf{y}} p(\mathbf{y} \mid \mathbf{x})$. In neural machine translation, we fit a parameterized model to maximize the conditional probability of sentence pairs using a parallel training corpus. Once the conditional distribution is learned by a translation model, given a source sentence a corresponding translation can be generated by searching for the sentence that maximizes the conditional probability.

Recently, a number of papers have proposed the use of neural networks to directly learn this conditional distribution (see, e.g., Kalchbrenner and Blunsom, 2013; Cho *et al.*, 2014a; Sutskever *et al.*, 2014; Cho *et al.*, 2014b; Forcada and Ñeco, 1997). This neural machine translation approach typically consists of two components, the first of which encodes a source sentence $\mathbf{x}$ and the second decodes to a target sentence $\mathbf{y}$. For instance, two recurrent neural networks (RNN) were used by (Cho *et al.*, 2014a) and (Sutskever *et al.*, 2014) to encode a variable-length source sentence into a fixed-length vector and to decode the vector into a variable-length target sentence.

Despite being a quite new approach, neural machine translation has already shown promising results. Sutskever *et al.* (2014) reported that the neural machine translation based on RNNs with long short-term memory (LSTM) units achieves close to the state-of-the-art performance of the conventional phrase-based machine translation system on an English-to-French translation task.[1] Adding neural components to existing translation systems, for instance, to score the phrase pairs in the phrase table (Cho *et al.*, 2014a) or to re-rank candidate translations (Sutskever *et al.*, 2014), has allowed to surpass the previous state-of-the-art performance level.

2.1 RNN Encoder–Decoder

Here, we describe briefly the underlying framework, called *RNN Encoder–Decoder*, proposed by Cho *et al.* (2014a) and Sutskever *et al.* (2014) upon which we build a novel architecture that learns to align and translate simultaneously.

In the Encoder–Decoder framework, an encoder reads the input sentence, a sequence of vectors $\mathbf{x} = (x_1, \cdots, x_{T_x})$, into a vector c.[2] The most common approach is to use an RNN such that

$$h_t = f(x_t, h_{t-1}) \tag{1}$$

and

$$c = q(\{h_1, \cdots, h_{T_x}\}),$$

where $h_t \in \mathbb{R}^n$ is a hidden state at time t, and c is a vector generated from the sequence of the hidden states. f and q are some nonlinear functions. Sutskever *et al.* (2014) used an LSTM as f and $q(\{h_1, \cdots, h_T\}) = h_T$, for instance.

[1] We mean by the state-of-the-art performance, the performance of the conventional phrase-based system without using any neural network-based component.

[2] Although most of the previous works (see, e.g., Cho *et al.*, 2014a; Sutskever *et al.*, 2014; Kalchbrenner and Blunsom, 2013) used to encode a variable-length input sentence into a *fixed-length* vector, it is not necessary, and even it may be beneficial to have a *variable-length* vector, as we will show later.

The decoder is often trained to predict the next word $y_{t'}$ given the context vector c and all the previously predicted words $\{y_1, \cdots, y_{t'-1}\}$. In other words, the decoder defines a probability over the translation $\mathbf{y}$ by decomposing the joint probability into the ordered conditionals:

$$p(\mathbf{y}) = \prod_{t=1}^{T} p(y_t \mid \{y_1, \cdots, y_{t-1}\}, c), \tag{2}$$

where $\mathbf{y} = \left(y_1, \cdots, y_{T_y}\right)$. With an RNN, each conditional probability is modeled as

$$p(y_t \mid \{y_1, \cdots, y_{t-1}\}, c) = g(y_{t-1}, s_t, c), \tag{3}$$

where g is a nonlinear, potentially multi-layered, function that outputs the probability of y_t, and s_t is the hidden state of the RNN. It should be noted that other architectures such as a hybrid of an RNN and a de-convolutional neural network can be used (Kalchbrenner and Blunsom, 2013).

3 Learning to Align and Translate

In this section, we propose a novel architecture for neural machine translation. The new architecture consists of a bidirectional RNN as an encoder (Sec. 3.2) and a decoder that emulates searching through a source sentence during decoding a translation (Sec. 3.1).

3.1 Decoder: General Description

In a new model architecture, we define each conditional probability in Eq. (2) as:

$$p(y_i | y_1, \ldots, y_{i-1}, \mathbf{x}) = g(y_{i-1}, s_i, c_i), \tag{4}$$

where s_i is an RNN hidden state for time i, computed by

$$s_i = f(s_{i-1}, y_{i-1}, c_i).$$

It should be noted that unlike the existing encoder–decoder approach (see Eq. (2)), here the probability is conditioned on a distinct context vector c_i for each target word y_i.

The context vector c_i depends on a sequence of *annotations* $(h_1, \cdots, h_{T_x})$ to which an encoder maps the input sentence. Each annotation h_i contains information about the whole input sequence with a strong focus on the parts surrounding the i-th word of the input sequence. We explain in detail how the annotations are computed in the next section.

The context vector c_i is, then, computed as a weighted sum of these annotations h_i:

$$c_i = \sum_{j=1}^{T_x} \alpha_{ij} h_j. \tag{5}$$

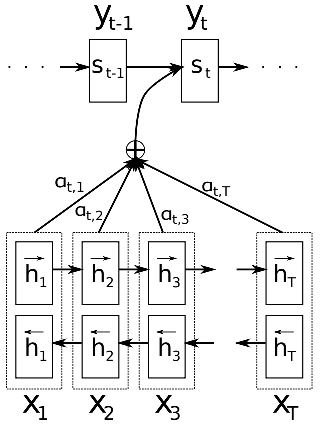

Figure 1: The graphical illustration of the proposed model trying to generate the t-th target word y_t given a source sentence $(x_1, x_2, \ldots, x_T)$.

The weight α_{ij} of each annotation h_j is computed by

$$\alpha_{ij} = \frac{\exp\left(e_{ij}\right)}{\sum_{k=1}^{T_x} \exp\left(e_{ik}\right)}, \tag{6}$$

where

$$e_{ij} = a(s_{i-1}, h_j)$$

is an *alignment model* which scores how well the inputs around position j and the output at position i match. The score is based on the RNN hidden state s_{i-1} (just before emitting y_i, Eq. (4)) and the j-th annotation h_j of the input sentence.

We parametrize the alignment model a as a feedforward neural network which is jointly trained with all the other components of the proposed system. Note that unlike in traditional machine translation,

the alignment is not considered to be a latent variable. Instead, the alignment model directly computes a soft alignment, which allows the gradient of the cost function to be backpropagated through. This gradient can be used to train the alignment model as well as the whole translation model jointly.

We can understand the approach of taking a weighted sum of all the annotations as computing an *expected annotation*, where the expectation is over possible alignments. Let α_{ij} be a probability that the target word y_i is aligned to, or translated from, a source word x_j. Then, the i-th context vector c_i is the expected annotation over all the annotations with probabilities α_{ij}.

The probability α_{ij}, or its associated energy e_{ij}, reflects the importance of the annotation h_j with respect to the previous hidden state s_{i-1} in deciding the next state s_i and generating y_i. Intuitively, this implements a mechanism of attention in the decoder. The decoder decides parts of the source sentence to pay attention to. By letting the decoder have an attention mechanism, we relieve the encoder from the burden of having to encode all information in the source sentence into a fixed-length vector. With this new approach the information can be spread throughout the sequence of annotations, which can be selectively retrieved by the decoder accordingly.

3.2 Encoder: Bidirectional RNN for Annotating Sequences

The usual RNN, described in Eq. (1), reads an input sequence $\mathbf{x}$ in order starting from the first symbol x_1 to the last one x_{T_x}. However, in the proposed scheme, we would like the annotation of each word to summarize not only the preceding words, but also the following words. Hence, we propose to use a bidirectional RNN (BiRNN, Schuster and Paliwal, 1997), which has been successfully used recently in speech recognition (see, e.g., Graves *et al.*, 2013).

A BiRNN consists of forward and backward RNN's. The forward RNN $\overrightarrow{f}$ reads the input sequence as it is ordered (from x_1 to x_{T_x}) and calculates a sequence of *forward hidden states* ($\overrightarrow{h}_1, \cdots, \overrightarrow{h}_{T_x}$). The backward RNN $\overleftarrow{f}$ reads the sequence in the reverse order (from x_{T_x} to x_1), resulting in a sequence of *backward hidden states* ($\overleftarrow{h}_1, \cdots, \overleftarrow{h}_{T_x}$).

We obtain an annotation for each word x_j by concatenating the forward hidden state $\overrightarrow{h}_j$ and the backward one $\overleftarrow{h}_j$, i.e., $h_j = \left[\overrightarrow{h}_j^\top; \overleftarrow{h}_j^\top\right]^\top$. In this way, the annotation h_j contains the summaries of both the preceding words and the following words. Due to the tendency of RNNs to better represent recent inputs, the annotation h_j will be focused on the words around x_j. This sequence of annotations is used by the decoder and the alignment model later to compute the context vector (Eqs. (5)–(6)).

See Fig. 1 for the graphical illustration of the proposed model.

4 Experiment Settings

We evaluate the proposed approach on the task of English-to-French translation. We use the bilingual, parallel corpora provided by ACL WMT '14.[3] As a comparison, we also report the performance of an RNN Encoder–Decoder which was proposed recently by Cho *et al.* (2014a). We use the same training procedures and the same dataset for both models.[4]

4.1 Dataset

WMT '14 contains the following English-French parallel corpora: Europarl (61M words), news commentary (5.5M), UN (421M) and two crawled corpora of 90M and 272.5M words respectively, totaling 850M words. Following the procedure described in Cho *et al.* (2014a), we reduce the size of the combined corpus to have 348M words using the data selection method by Axelrod *et al.* (2011).[5] We do not use any monolingual data other than the mentioned parallel corpora, although it may be possible to use a much larger monolingual corpus to pretrain an encoder. We concatenate news-test-

[3] `http://www.statmt.org/wmt14/translation-task.html`

[4] Implementations are available at `https://github.com/lisa-groundhog/GroundHog`.

[5] Available online at `http://www-lium.univ-lemans.fr/~schwenk/cslm_joint_paper/`.

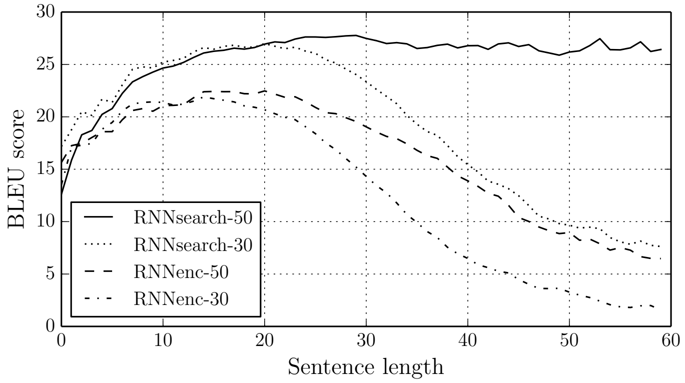

Figure 2: The BLEU scores of the generated translations on the test set with respect to the lengths of the sentences. The results are on the full test set which includes sentences having unknown words to the models.

2012 and news-test-2013 to make a development (validation) set, and evaluate the models on the test set (news-test-2014) from WMT '14, which consists of 3003 sentences not present in the training data.

After a usual tokenization[6], we use a shortlist of 30,000 most frequent words in each language to train our models. Any word not included in the shortlist is mapped to a special token ([UNK]). We do not apply any other special preprocessing, such as lowercasing or stemming, to the data.

4.2 MODELS

We train two types of models. The first one is an RNN Encoder–Decoder (RNNencdec, Cho *et al.*, 2014a), and the other is the proposed model, to which we refer as RNNsearch. We train each model twice: first with the sentences of length up to 30 words (RNNencdec-30, RNNsearch-30) and then with the sentences of length up to 50 word (RNNencdec-50, RNNsearch-50).

The encoder and decoder of the RNNencdec have 1000 hidden units each.[7] The encoder of the RNNsearch consists of forward and backward recurrent neural networks (RNN) each having 1000 hidden units. Its decoder has 1000 hidden units. In both cases, we use a multilayer network with a single maxout (Goodfellow *et al.*, 2013) hidden layer to compute the conditional probability of each target word (Pascanu *et al.*, 2014).

We use a minibatch stochastic gradient descent (SGD) algorithm together with Adadelta (Zeiler, 2012) to train each model. Each SGD update direction is computed using a minibatch of 80 sentences. We trained each model for approximately 5 days.

Once a model is trained, we use a beam search to find a translation that approximately maximizes the conditional probability (see, e.g., Graves, 2012; Boulanger-Lewandowski *et al.*, 2013). Sutskever *et al.* (2014) used this approach to generate translations from their neural machine translation model.

For more details on the architectures of the models and training procedure used in the experiments, see Appendices A and B.

5 RESULTS

5.1 QUANTITATIVE RESULTS

In Table 1, we list the translation performances measured in BLEU score. It is clear from the table that in all the cases, the proposed RNNsearch outperforms the conventional RNNencdec. More importantly, the performance of the RNNsearch is as high as that of the conventional phrase-based translation system (Moses), when only the sentences consisting of known words are considered. This is a significant achievement, considering that Moses uses a separate monolingual corpus (418M words) in addition to the parallel corpora we used to train the RNNsearch and RNNencdec.

[6] We used the tokenization script from the open-source machine translation package, Moses.

[7] In this paper, by a 'hidden unit', we always mean the gated hidden unit (see Appendix A.1.1).

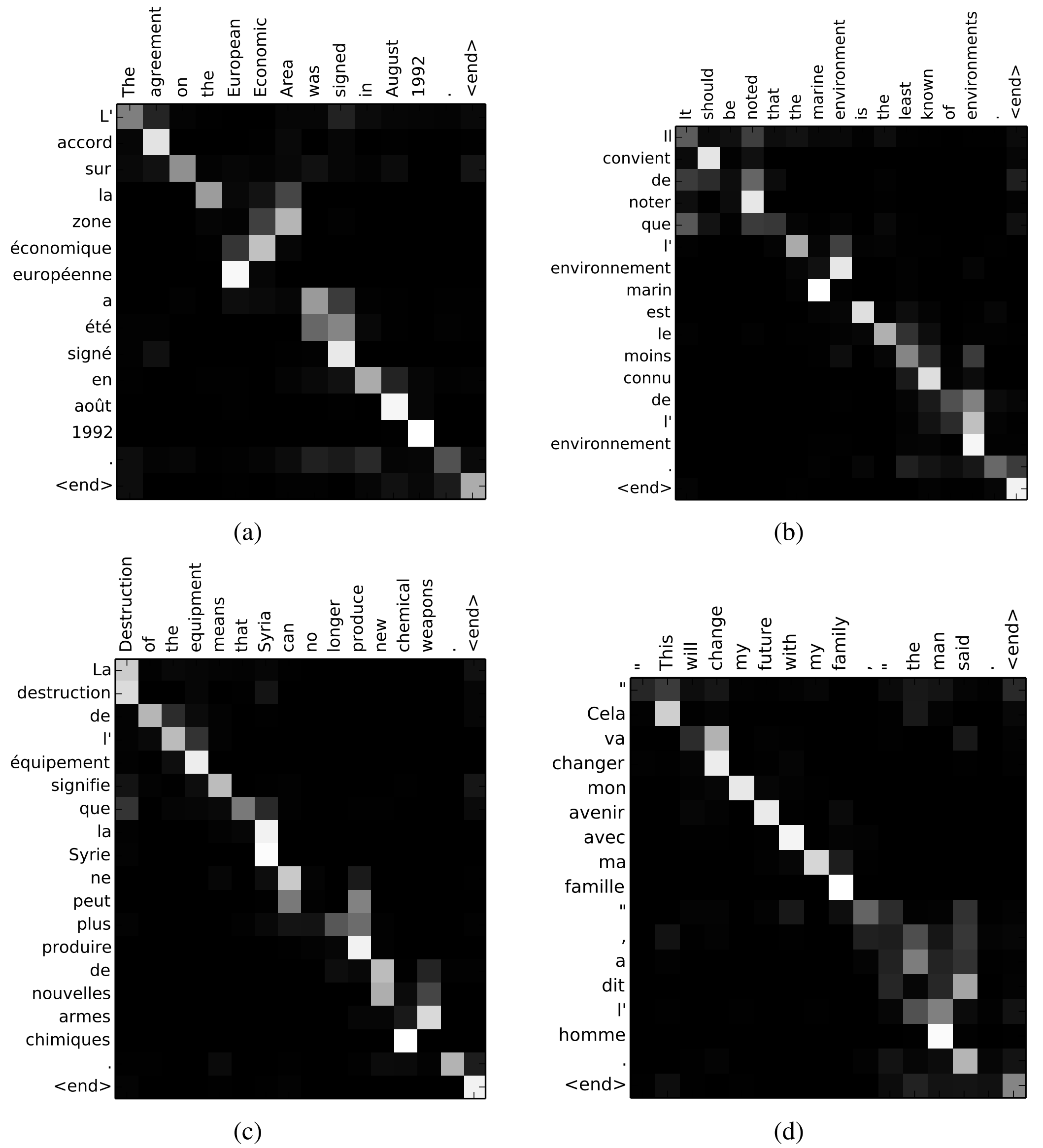

Figure 3: Four sample alignments found by RNNsearch-50. The x-axis and y-axis of each plot correspond to the words in the source sentence (English) and the generated translation (French), respectively. Each pixel shows the weight α_{ij} of the annotation of the j-th source word for the i-th target word (see Eq. (6)), in grayscale (0: black, 1: white). (a) an arbitrary sentence. (b–d) three randomly selected samples among the sentences without any unknown words and of length between 10 and 20 words from the test set.

One of the motivations behind the proposed approach was the use of a fixed-length context vector in the basic encoder–decoder approach. We conjectured that this limitation may make the basic encoder–decoder approach to underperform with long sentences. In Fig. 2, we see that the performance of RNNencdec dramatically drops as the length of the sentences increases. On the other hand, both RNNsearch-30 and RNNsearch-50 are more robust to the length of the sentences. RNNsearch-50, especially, shows no performance deterioration even with sentences of length 50 or more. This superiority of the proposed model over the basic encoder–decoder is further confirmed by the fact that the RNNsearch-30 even outperforms RNNencdec-50 (see Table 1).

Model	All	No UNK°
RNNencdec-30	13.93	24.19
RNNsearch-30	21.50	31.44
RNNencdec-50	17.82	26.71
RNNsearch-50	26.75	34.16
RNNsearch-50⋆	28.45	36.15
Moses	33.30	35.63

Table 1: BLEU scores of the trained models computed on the test set. The second and third columns show respectively the scores on all the sentences and, on the sentences without any unknown word in themselves and in the reference translations. Note that RNNsearch-50⋆ was trained much longer until the performance on the development set stopped improving. (◦) We disallowed the models to generate [UNK] tokens when only the sentences having no unknown words were evaluated (last column).

5.2 Qualitative Analysis

5.2.1 Alignment

The proposed approach provides an intuitive way to inspect the (soft-)alignment between the words in a generated translation and those in a source sentence. This is done by visualizing the annotation weights α_{ij} from Eq. (6), as in Fig. 3. Each row of a matrix in each plot indicates the weights associated with the annotations. From this we see which positions in the source sentence were considered more important when generating the target word.

We can see from the alignments in Fig. 3 that the alignment of words between English and French is largely monotonic. We see strong weights along the diagonal of each matrix. However, we also observe a number of non-trivial, non-monotonic alignments. Adjectives and nouns are typically ordered differently between French and English, and we see an example in Fig. 3 (a). From this figure, we see that the model correctly translates a phrase [European Economic Area] into [zone économique européen]. The RNNsearch was able to correctly align [zone] with [Area], jumping over the two words ([European] and [Economic]), and then looked one word back at a time to complete the whole phrase [zone économique européenne].

The strength of the soft-alignment, opposed to a hard-alignment, is evident, for instance, from Fig. 3 (d). Consider the source phrase [the man] which was translated into [l' homme]. Any hard alignment will map [the] to [l'] and [man] to [homme]. This is not helpful for translation, as one must consider the word following [the] to determine whether it should be translated into [le], [la], [les] or [l']. Our soft-alignment solves this issue naturally by letting the model look at both [the] and [man], and in this example, we see that the model was able to correctly translate [the] into [l']. We observe similar behaviors in all the presented cases in Fig. 3. An additional benefit of the soft alignment is that it naturally deals with source and target phrases of different lengths, without requiring a counter-intuitive way of mapping some words to or from nowhere ([NULL]) (see, e.g., Chapters 4 and 5 of Koehn, 2010).

5.2.2 Long Sentences

As clearly visible from Fig. 2 the proposed model (RNNsearch) is much better than the conventional model (RNNencdec) at translating long sentences. This is likely due to the fact that the RNNsearch does not require encoding a long sentence into a fixed-length vector perfectly, but only accurately encoding the parts of the input sentence that surround a particular word.

As an example, consider this source sentence from the test set:

> *An admitting privilege is the right of a doctor to admit a patient to a hospital or a medical centre <u>to carry out a diagnosis or a procedure, based on his status as a health care worker at a hospital.</u>*

The RNNencdec-50 translated this sentence into:

> *Un privilège d'admission est le droit d'un médecin de reconnaître un patient à l'hôpital ou un centre médical <u>d'un diagnostic ou de prendre un diagnostic en fonction de son état de santé.</u>*

The RNNencdec-50 correctly translated the source sentence until [a medical center]. However, from there on (underlined), it deviated from the original meaning of the source sentence. For instance, it replaced [based on his status as a health care worker at a hospital] in the source sentence with [en fonction de son état de santé] ("based on his state of health").

On the other hand, the RNNsearch-50 generated the following correct translation, preserving the whole meaning of the input sentence without omitting any details:

> *Un privilège d'admission est le droit d'un médecin d'admettre un patient à un hôpital ou un centre médical pour effectuer un diagnostic ou une procédure, selon son statut de travailleur des soins de santé à l'hôpital.*

Let us consider another sentence from the test set:

> *This kind of experience is part of Disney's efforts to "extend the lifetime of its series and build new relationships with audiences via digital platforms that are becoming ever more important," he added.*

The translation by the RNNencdec-50 is

> *Ce type d'expérience fait partie des initiatives du Disney pour "prolonger la durée de vie de ses nouvelles et de développer des liens avec les lecteurs numériques qui deviennent plus complexes.*

As with the previous example, the RNNencdec began deviating from the actual meaning of the source sentence after generating approximately 30 words (see the underlined phrase). After that point, the quality of the translation deteriorates, with basic mistakes such as the lack of a closing quotation mark.

Again, the RNNsearch-50 was able to translate this long sentence correctly:

> *Ce genre d'expérience fait partie des efforts de Disney pour "prolonger la durée de vie de ses séries et créer de nouvelles relations avec des publics via des plateformes numériques de plus en plus importantes", a-t-il ajouté.*

In conjunction with the quantitative results presented already, these qualitative observations confirm our hypotheses that the RNNsearch architecture enables far more reliable translation of long sentences than the standard RNNencdec model.

In Appendix C, we provide a few more sample translations of long source sentences generated by the RNNencdec-50, RNNsearch-50 and Google Translate along with the reference translations.

6 Related Work

6.1 Learning to Align

A similar approach of aligning an output symbol with an input symbol was proposed recently by Graves (2013) in the context of handwriting synthesis. Handwriting synthesis is a task where the model is asked to generate handwriting of a given sequence of characters. In his work, he used a mixture of Gaussian kernels to compute the weights of the annotations, where the location, width and mixture coefficient of each kernel was predicted from an alignment model. More specifically, his alignment was restricted to predict the location such that the location increases monotonically.

The main difference from our approach is that, in (Graves, 2013), the modes of the weights of the annotations only move in one direction. In the context of machine translation, this is a severe limitation, as (long-distance) reordering is often needed to generate a grammatically correct translation (for instance, English-to-German).

Our approach, on the other hand, requires computing the annotation weight of every word in the source sentence for each word in the translation. This drawback is not severe with the task of translation in which most of input and output sentences are only 15–40 words. However, this may limit the applicability of the proposed scheme to other tasks.

6.2 Neural Networks for Machine Translation

Since Bengio *et al.* (2003) introduced a neural probabilistic language model which uses a neural network to model the conditional probability of a word given a fixed number of the preceding words, neural networks have widely been used in machine translation. However, the role of neural networks has been largely limited to simply providing a single feature to an existing statistical machine translation system or to re-rank a list of candidate translations provided by an existing system.

For instance, Schwenk (2012) proposed using a feedforward neural network to compute the score of a pair of source and target phrases and to use the score as an additional feature in the phrase-based statistical machine translation system. More recently, Kalchbrenner and Blunsom (2013) and Devlin *et al.* (2014) reported the successful use of the neural networks as a sub-component of the existing translation system. Traditionally, a neural network trained as a target-side language model has been used to rescore or rerank a list of candidate translations (see, e.g., Schwenk *et al.*, 2006).

Although the above approaches were shown to improve the translation performance over the state-of-the-art machine translation systems, we are more interested in a more ambitious objective of designing a completely new translation system based on neural networks. The neural machine translation approach we consider in this paper is therefore a radical departure from these earlier works. Rather than using a neural network as a part of the existing system, our model works on its own and generates a translation from a source sentence directly.

7 Conclusion

The conventional approach to neural machine translation, called an encoder–decoder approach, encodes a whole input sentence into a fixed-length vector from which a translation will be decoded. We conjectured that the use of a fixed-length context vector is problematic for translating long sentences, based on a recent empirical study reported by Cho *et al.* (2014b) and Pouget-Abadie *et al.* (2014).

In this paper, we proposed a novel architecture that addresses this issue. We extended the basic encoder–decoder by letting a model (soft-)search for a set of input words, or their annotations computed by an encoder, when generating each target word. This frees the model from having to encode a whole source sentence into a fixed-length vector, and also lets the model focus only on information relevant to the generation of the next target word. This has a major positive impact on the ability of the neural machine translation system to yield good results on longer sentences. Unlike with the traditional machine translation systems, all of the pieces of the translation system, including the alignment mechanism, are jointly trained towards a better log-probability of producing correct translations.

We tested the proposed model, called RNNsearch, on the task of English-to-French translation. The experiment revealed that the proposed RNNsearch outperforms the conventional encoder–decoder model (RNNencdec) significantly, regardless of the sentence length and that it is much more robust to the length of a source sentence. From the qualitative analysis where we investigated the (soft-)alignment generated by the RNNsearch, we were able to conclude that the model can correctly align each target word with the relevant words, or their annotations, in the source sentence as it generated a correct translation.

Perhaps more importantly, the proposed approach achieved a translation performance comparable to the existing phrase-based statistical machine translation. It is a striking result, considering that the proposed architecture, or the whole family of neural machine translation, has only been proposed as recently as this year. We believe the architecture proposed here is a promising step toward better machine translation and a better understanding of natural languages in general.

One of challenges left for the future is to better handle unknown, or rare words. This will be required for the model to be more widely used and to match the performance of current state-of-the-art machine translation systems in all contexts.

Acknowledgments

The authors would like to thank the developers of Theano (Bergstra *et al.*, 2010; Bastien *et al.*, 2012). We acknowledge the support of the following agencies for research funding and computing support: NSERC, Calcul Québec, Compute Canada, the Canada Research Chairs and CIFAR. Bahdanau thanks the support from Planet Intelligent Systems GmbH. We also thank Felix Hill, Bart van Merriénboer, Jean Pouget-Abadie, Coline Devin and Tae-Ho Kim.

References

Axelrod, A., He, X., and Gao, J. (2011). Domain adaptation via pseudo in-domain data selection. In *Proceedings of the ACL Conference on Empirical Methods in Natural Language Processing (EMNLP)*, pages 355–362. Association for Computational Linguistics.

Bastien, F., Lamblin, P., Pascanu, R., Bergstra, J., Goodfellow, I. J., Bergeron, A., Bouchard, N., and Bengio, Y. (2012). Theano: new features and speed improvements. Deep Learning and Unsupervised Feature Learning NIPS 2012 Workshop.

Bengio, Y., Simard, P., and Frasconi, P. (1994). Learning long-term dependencies with gradient descent is difficult. *IEEE Transactions on Neural Networks*, **5**(2), 157–166.

Bengio, Y., Ducharme, R., Vincent, P., and Janvin, C. (2003). A neural probabilistic language model. *J. Mach. Learn. Res.*, **3**, 1137–1155.

Bergstra, J., Breuleux, O., Bastien, F., Lamblin, P., Pascanu, R., Desjardins, G., Turian, J., Warde-Farley, D., and Bengio, Y. (2010). Theano: a CPU and GPU math expression compiler. In *Proceedings of the Python for Scientific Computing Conference (SciPy)*. Oral Presentation.

Boulanger-Lewandowski, N., Bengio, Y., and Vincent, P. (2013). Audio chord recognition with recurrent neural networks. In *ISMIR*.

Cho, K., van Merrienboer, B., Gulcehre, C., Bougares, F., Schwenk, H., and Bengio, Y. (2014a). Learning phrase representations using RNN encoder-decoder for statistical machine translation. In *Proceedings of the Empiricial Methods in Natural Language Processing (EMNLP 2014)*. to appear.

Cho, K., van Merriënboer, B., Bahdanau, D., and Bengio, Y. (2014b). On the properties of neural machine translation: Encoder–Decoder approaches. In *Eighth Workshop on Syntax, Semantics and Structure in Statistical Translation*. to appear.

Devlin, J., Zbib, R., Huang, Z., Lamar, T., Schwartz, R., and Makhoul, J. (2014). Fast and robust neural network joint models for statistical machine translation. In *Association for Computational Linguistics*.

Forcada, M. L. and Ñeco, R. P. (1997). Recursive hetero-associative memories for translation. In J. Mira, R. Moreno-Díaz, and J. Cabestany, editors, *Biological and Artificial Computation: From Neuroscience to Technology*, volume 1240 of *Lecture Notes in Computer Science*, pages 453–462. Springer Berlin Heidelberg.

Goodfellow, I., Warde-Farley, D., Mirza, M., Courville, A., and Bengio, Y. (2013). Maxout networks. In *Proceedings of The 30th International Conference on Machine Learning*, pages 1319–1327.

Graves, A. (2012). Sequence transduction with recurrent neural networks. In *Proceedings of the 29th International Conference on Machine Learning (ICML 2012)*.

Graves, A. (2013). Generating sequences with recurrent neural networks. *arXiv:*`1308.0850 [cs.NE]`.

Graves, A., Jaitly, N., and Mohamed, A.-R. (2013). Hybrid speech recognition with deep bidirectional LSTM. In *Automatic Speech Recognition and Understanding (ASRU), 2013 IEEE Workshop on*, pages 273–278.

Hermann, K. and Blunsom, P. (2014). Multilingual distributed representations without word alignment. In *Proceedings of the Second International Conference on Learning Representations (ICLR 2014)*.

Hochreiter, S. (1991). Untersuchungen zu dynamischen neuronalen Netzen. Diploma thesis, Institut für Informatik, Lehrstuhl Prof. Brauer, Technische Universität München.

Hochreiter, S. and Schmidhuber, J. (1997). Long short-term memory. *Neural Computation*, **9**(8), 1735–1780.

Kalchbrenner, N. and Blunsom, P. (2013). Recurrent continuous translation models. In *Proceedings of the ACL Conference on Empirical Methods in Natural Language Processing (EMNLP)*, pages 1700–1709. Association for Computational Linguistics.

Koehn, P. (2010). *Statistical Machine Translation*. Cambridge University Press, New York, NY, USA.

Koehn, P., Och, F. J., and Marcu, D. (2003). Statistical phrase-based translation. In *Proceedings of the 2003 Conference of the North American Chapter of the Association for Computational Linguistics on Human Language Technology - Volume 1*, NAACL '03, pages 48–54, Stroudsburg, PA, USA. Association for Computational Linguistics.

Pascanu, R., Mikolov, T., and Bengio, Y. (2013a). On the difficulty of training recurrent neural networks. In *ICML'2013*.

Pascanu, R., Mikolov, T., and Bengio, Y. (2013b). On the difficulty of training recurrent neural networks. In *Proceedings of the 30th International Conference on Machine Learning (ICML 2013)*.

Pascanu, R., Gulcehre, C., Cho, K., and Bengio, Y. (2014). How to construct deep recurrent neural networks. In *Proceedings of the Second International Conference on Learning Representations (ICLR 2014)*.

Pouget-Abadie, J., Bahdanau, D., van Merriënboer, B., Cho, K., and Bengio, Y. (2014). Overcoming the curse of sentence length for neural machine translation using automatic segmentation. In *Eighth Workshop on Syntax, Semantics and Structure in Statistical Translation*. to appear.

Schuster, M. and Paliwal, K. K. (1997). Bidirectional recurrent neural networks. *Signal Processing, IEEE Transactions on*, **45**(11), 2673–2681.

Schwenk, H. (2012). Continuous space translation models for phrase-based statistical machine translation. In M. Kay and C. Boitet, editors, *Proceedings of the 24th International Conference on Computational Linguistics (COLIN)*, pages 1071–1080. Indian Institute of Technology Bombay.

Schwenk, H., Dchelotte, D., and Gauvain, J.-L. (2006). Continuous space language models for statistical machine translation. In *Proceedings of the COLING/ACL on Main conference poster sessions*, pages 723–730. Association for Computational Linguistics.

Sutskever, I., Vinyals, O., and Le, Q. (2014). Sequence to sequence learning with neural networks. In *Advances in Neural Information Processing Systems (NIPS 2014)*.

Zeiler, M. D. (2012). ADADELTA: An adaptive learning rate method. *arXiv:*`1212.5701 [cs.LG]`.

A MODEL ARCHITECTURE

A.1 ARCHITECTURAL CHOICES

The proposed scheme in Section 3 is a general framework where one can freely define, for instance, the activation functions f of recurrent neural networks (RNN) and the alignment model a. Here, we describe the choices we made for the experiments in this paper.

A.1.1 RECURRENT NEURAL NETWORK

For the activation function f of an RNN, we use the gated hidden unit recently proposed by Cho *et al.* (2014a). The gated hidden unit is an alternative to the conventional *simple* units such as an element-wise $\tanh$. This gated unit is similar to a long short-term memory (LSTM) unit proposed earlier by Hochreiter and Schmidhuber (1997), sharing with it the ability to better model and learn long-term dependencies. This is made possible by having computation paths in the unfolded RNN for which the product of derivatives is close to 1. These paths allow gradients to flow backward easily without suffering too much from the vanishing effect (Hochreiter, 1991; Bengio *et al.*, 1994; Pascanu *et al.*, 2013a). It is therefore possible to use LSTM units instead of the gated hidden unit described here, as was done in a similar context by Sutskever *et al.* (2014).

The new state s_i of the RNN employing n gated hidden units[8] is computed by

$$s_i = f(s_{i-1}, y_{i-1}, c_i) = (1 - z_i) \circ s_{i-1} + z_i \circ \tilde{s}_i,$$

where $\circ$ is an element-wise multiplication, and z_i is the output of the update gates (see below). The proposed updated state $\tilde{s}_i$ is computed by

$$\tilde{s}_i = \tanh\left(We(y_{i-1}) + U\left[r_i \circ s_{i-1}\right] + Cc_i\right),$$

where $e(y_{i-1}) \in \mathbb{R}^m$ is an m-dimensional embedding of a word y_{i-1}, and r_i is the output of the reset gates (see below). When y_i is represented as a 1-of-K vector, $e(y_i)$ is simply a column of an embedding matrix $E \in \mathbb{R}^{m \times K}$. Whenever possible, we omit bias terms to make the equations less cluttered.

The update gates z_i allow each hidden unit to maintain its previous activation, and the reset gates r_i control how much and what information from the previous state should be reset. We compute them by

$$z_i = \sigma\left(W_z e(y_{i-1}) + U_z s_{i-1} + C_z c_i\right),$$
$$r_i = \sigma\left(W_r e(y_{i-1}) + U_r s_{i-1} + C_r c_i\right),$$

where $\sigma(\cdot)$ is a logistic sigmoid function.

At each step of the decoder, we compute the output probability (Eq. (4)) as a multi-layered function (Pascanu *et al.*, 2014). We use a single hidden layer of maxout units (Goodfellow *et al.*, 2013) and normalize the output probabilities (one for each word) with a softmax function (see Eq. (6)).

A.1.2 ALIGNMENT MODEL

The alignment model should be designed considering that the model needs to be evaluated $T_x \times T_y$ times for each sentence pair of lengths T_x and T_y. In order to reduce computation, we use a single-layer multilayer perceptron such that

$$a(s_{i-1}, h_j) = v_a^\top \tanh\left(W_a s_{i-1} + U_a h_j\right),$$

where $W_a \in \mathbb{R}^{n \times n}, U_a \in \mathbb{R}^{n \times 2n}$ and $v_a \in \mathbb{R}^n$ are the weight matrices. Since $U_a h_j$ does not depend on i, we can pre-compute it in advance to minimize the computational cost.

[8] Here, we show the formula of the decoder. The same formula can be used in the encoder by simply ignoring the context vector c_i and the related terms.

A.2 Detailed Description of the Model

A.2.1 Encoder

In this section, we describe in detail the architecture of the proposed model (RNNsearch) used in the experiments (see Sec. 4–5). From here on, we omit all bias terms in order to increase readability.

The model takes a source sentence of 1-of-K coded word vectors as input

$$\mathbf{x} = (x_1, \ldots, x_{T_x}),\ x_i \in \mathbb{R}^{K_x}$$

and outputs a translated sentence of 1-of-K coded word vectors

$$\mathbf{y} = (y_1, \ldots, y_{T_y}),\ y_i \in \mathbb{R}^{K_y},$$

where K_x and K_y are the vocabulary sizes of source and target languages, respectively. T_x and T_y respectively denote the lengths of source and target sentences.

First, the forward states of the bidirectional recurrent neural network (BiRNN) are computed:

$$\overrightarrow{h}_i = \begin{cases} (1 - \overrightarrow{z}_i) \circ \overrightarrow{h}_{i-1} + \overrightarrow{z}_i \circ \underline{\overrightarrow{h}}_i & \text{, if } i > 0 \\ 0 & \text{, if } i = 0 \end{cases}$$

where

$$\begin{aligned} \underline{\overrightarrow{h}}_i =& \tanh\left(\overrightarrow{W}\overline{E}x_i + \overrightarrow{U}\left[\overrightarrow{r}_i \circ \overrightarrow{h}_{i-1}\right]\right) \\ \overrightarrow{z}_i =& \sigma\left(\overrightarrow{W}_z\overline{E}x_i + \overrightarrow{U}_z\overrightarrow{h}_{i-1}\right) \\ \overrightarrow{r}_i =& \sigma\left(\overrightarrow{W}_r\overline{E}x_i + \overrightarrow{U}_r\overrightarrow{h}_{i-1}\right). \end{aligned}$$

$\overline{E} \in \mathbb{R}^{m \times K_x}$ is the word embedding matrix. $\overrightarrow{W}, \overrightarrow{W}_z, \overrightarrow{W}_r \in \mathbb{R}^{n \times m}$, $\overrightarrow{U}, \overrightarrow{U}_z, \overrightarrow{U}_r \in \mathbb{R}^{n \times n}$ are weight matrices. m and n are the word embedding dimensionality and the number of hidden units, respectively. $\sigma(\cdot)$ is as usual a logistic sigmoid function.

The backward states $(\overleftarrow{h}_1, \cdots, \overleftarrow{h}_{T_x})$ are computed similarly. We share the word embedding matrix $\overline{E}$ between the forward and backward RNNs, unlike the weight matrices.

We concatenate the forward and backward states to to obtain the annotations $(h_1, h_2, \cdots, h_{T_x})$, where

$$h_i = \left[\begin{array}{c} \overrightarrow{h}_i \\ \overleftarrow{h}_i \end{array}\right] \tag{7}$$

A.2.2 Decoder

The hidden state s_i of the decoder given the annotations from the encoder is computed by

$$s_i = (1 - z_i) \circ s_{i-1} + z_i \circ \tilde{s}_i,$$

where

$$\begin{aligned} \tilde{s}_i =& \tanh\left(WEy_{i-1} + U\left[r_i \circ s_{i-1}\right] + Cc_i\right) \\ z_i =& \sigma\left(W_zEy_{i-1} + U_zs_{i-1} + C_zc_i\right) \\ r_i =& \sigma\left(W_rEy_{i-1} + U_rs_{i-1} + C_rc_i\right) \end{aligned}$$

E is the word embedding matrix for the target language. $W, W_z, W_r \in \mathbb{R}^{n \times m}$, $U, U_z, U_r \in \mathbb{R}^{n \times n}$, and $C, C_z, C_r \in \mathbb{R}^{n \times 2n}$ are weights. Again, m and n are the word embedding dimensionality and the number of hidden units, respectively. The initial hidden state s_0 is computed by $s_0 = \tanh\left(W_s\overleftarrow{h}_1\right)$, where $W_s \in \mathbb{R}^{n \times n}$.

The context vector c_i are recomputed at each step by the alignment model:

$$c_i = \sum_{j=1}^{T_x} \alpha_{ij} h_j,$$

Model	Updates ($\times 10^5$)	Epochs	Hours	GPU	Train NLL	Dev. NLL
RNNenc-30	8.46	6.4	109	TITAN BLACK	28.1	53.0
RNNenc-50	6.00	4.5	108	Quadro K-6000	44.0	43.6
RNNsearch-30	4.71	3.6	113	TITAN BLACK	26.7	47.2
RNNsearch-50	2.88	2.2	111	Quadro K-6000	40.7	38.1
RNNsearch-50*	6.67	5.0	252	Quadro K-6000	36.7	35.2

Table 2: Learning statistics and relevant information. Each update corresponds to updating the parameters once using a single minibatch. One epoch is one pass through the training set. NLL is the average conditional log-probabilities of the sentences in either the training set or the development set. Note that the lengths of the sentences differ.

where

$$\alpha_{ij} = \frac{\exp(e_{ij})}{\sum_{k=1}^{T_x} \exp(e_{ik})}$$
$$e_{ij} = v_a^\top \tanh(W_a s_{i-1} + U_a h_j),$$

and h_j is the j-th annotation in the source sentence (see Eq. (7)). $v_a \in \mathbb{R}^{n'}, W_a \in \mathbb{R}^{n' \times n}$ and $U_a \in \mathbb{R}^{n' \times 2n}$ are weight matrices. Note that the model becomes RNN Encoder–Decoder (Cho *et al.*, 2014a), if we fix c_i to $\overrightarrow{h}_{T_x}$.

With the decoder state s_{i-1}, the context c_i and the last generated word y_{i-1}, we define the probability of a target word y_i as

$$p(y_i | s_i, y_{i-1}, c_i) \propto \exp\left(y_i^\top W_o t_i\right),$$

where

$$t_i = \left[\max\left\{\tilde{t}_{i,2j-1}, \tilde{t}_{i,2j}\right\}\right]_{j=1,\ldots,l}^\top$$

and $\tilde{t}_{i,k}$ is the k-th element of a vector $\tilde{t}_i$ which is computed by

$$\tilde{t}_i = U_o s_{i-1} + V_o E y_{i-1} + C_o c_i.$$

$W_o \in \mathbb{R}^{K_y \times l}$, $U_o \in \mathbb{R}^{2l \times n}$, $V_o \in \mathbb{R}^{2l \times m}$ and $C_o \in \mathbb{R}^{2l \times 2n}$ are weight matrices. This can be understood as having a deep output (Pascanu *et al.*, 2014) with a single maxout hidden layer (Goodfellow *et al.*, 2013).

A.2.3 Model Size

For all the models used in this paper, the size of a hidden layer n is 1000, the word embedding dimensionality m is 620 and the size of the maxout hidden layer in the deep output l is 500. The number of hidden units in the alignment model n' is 1000.

B Training Procedure

B.1 Parameter Initialization

We initialized the recurrent weight matrices $U, U_z, U_r, \overleftarrow{U}, \overleftarrow{U}_z, \overleftarrow{U}_r, \overrightarrow{U}, \overrightarrow{U}_z$ and $\overrightarrow{U}_r$ as random orthogonal matrices. For W_a and U_a, we initialized them by sampling each element from the Gaussian distribution of mean 0 and variance 0.001^2. All the elements of V_a and all the bias vectors were initialized to zero. Any other weight matrix was initialized by sampling from the Gaussian distribution of mean 0 and variance 0.01^2.

B.2 Training

We used the stochastic gradient descent (SGD) algorithm. Adadelta (Zeiler, 2012) was used to automatically adapt the learning rate of each parameter ($\epsilon = 10^{-6}$ and $\rho = 0.95$). We explicitly

normalized the L_2-norm of the gradient of the cost function each time to be at most a predefined threshold of 1, when the norm was larger than the threshold (Pascanu *et al.*, 2013b). Each SGD update direction was computed with a minibatch of 80 sentences.

At each update our implementation requires time proportional to the length of the longest sentence in a minibatch. Hence, to minimize the waste of computation, before every 20-th update, we retrieved 1600 sentence pairs, sorted them according to the lengths and split them into 20 minibatches. The training data was shuffled once before training and was traversed sequentially in this manner.

In Tables 2 we present the statistics related to training all the models used in the experiments.

C TRANSLATIONS OF LONG SENTENCES

Source	An admitting privilege is the right of a doctor to admit a patient to a hospital or a medical centre to carry out a diagnosis or a procedure, based on his status as a health care worker at a hospital.
Reference	Le privilège d'admission est le droit d'un médecin, en vertu de son statut de membre soignant d'un hôpital, d'admettre un patient dans un hôpital ou un centre médical afin d'y délivrer un diagnostic ou un traitement.
RNNenc-50	Un privilège d'admission est le droit d'un médecin de reconnaître un patient à l'hôpital ou un centre médical d'un diagnostic ou de prendre un diagnostic en fonction de son état de santé.
RNNsearch-50	Un privilège d'admission est le droit d'un médecin d'admettre un patient à un hôpital ou un centre médical pour effectuer un diagnostic ou une procédure, selon son statut de travailleur des soins de santé à l'hôpital.
Google Translate	Un privilège admettre est le droit d'un médecin d'admettre un patient dans un hôpital ou un centre médical pour effectuer un diagnostic ou une procédure, fondée sur sa situation en tant que travailleur de soins de santé dans un hôpital.

Source	This kind of experience is part of Disney's efforts to "extend the lifetime of its series and build new relationships with audiences via digital platforms that are becoming ever more important," he added.
Reference	Ce type d'expérience entre dans le cadre des efforts de Disney pour "étendre la durée de vie de ses séries et construire de nouvelles relations avec son public grâce à des plateformes numériques qui sont de plus en plus importantes", a-t-il ajouté.
RNNenc-50	Ce type d'expérience fait partie des initiatives du Disney pour "prolonger la durée de vie de ses nouvelles et de développer des liens avec les lecteurs numériques qui deviennent plus complexes.
RNNsearch-50	Ce genre d'expérience fait partie des efforts de Disney pour "prolonger la durée de vie de ses séries et créer de nouvelles relations avec des publics via des plateformes numériques de plus en plus importantes", a-t-il ajouté.
Google Translate	Ce genre d'expérience fait partie des efforts de Disney à "étendre la durée de vie de sa série et construire de nouvelles relations avec le public par le biais des plates-formes numériques qui deviennent de plus en plus important", at-il ajouté.

Source	In a press conference on Thursday, Mr Blair stated that there was nothing in this video that might constitute a "reasonable motive" that could lead to criminal charges being brought against the mayor.
Reference	En conférence de presse, jeudi, M. Blair a affirmé qu'il n'y avait rien dans cette vidéo qui puisse constituer des "motifs raisonnables" pouvant mener au dépôt d'une accusation criminelle contre le maire.
RNNenc-50	Lors de la conférence de presse de jeudi, M. Blair a dit qu'il n'y avait rien dans cette vidéo qui pourrait constituer une "motivation raisonnable" pouvant entraîner des accusations criminelles portées contre le maire.
RNNsearch-50	Lors d'une conférence de presse jeudi, M. Blair a déclaré qu'il n'y avait rien dans cette vidéo qui pourrait constituer un "motif raisonnable" qui pourrait conduire à des accusations criminelles contre le maire.
Google Translate	Lors d'une conférence de presse jeudi, M. Blair a déclaré qu'il n'y avait rien dans cette vido qui pourrait constituer un "motif raisonnable" qui pourrait mener à des accusations criminelles portes contre le maire.

Table 3: The translations generated by RNNenc-50 and RNNsearch-50 from long source sentences (30 words or more) selected from the test set. For each source sentence, we also show the gold-standard translation. The translations by Google Translate were made on 27 August 2014.
