# Supplementary figures and images for: Designing an intelligent push model for user emotional topics based on dynamic text categorization in social media news dissemination
Source: PeerJ Comput Sci. 2024 Dec 19;10:e2607. doi: 10.7717/peerj-cs.2607 (PMC11784813; doi:10.7717/peerj-cs.2607)

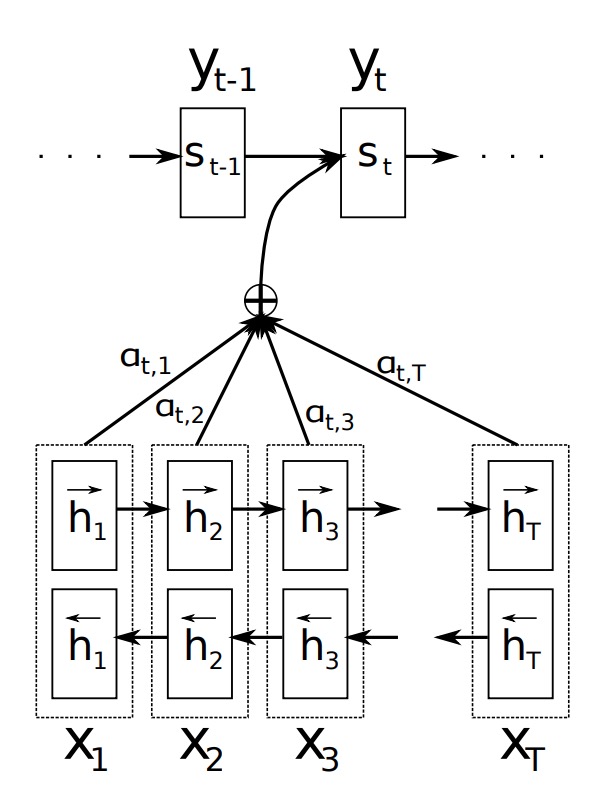

Supplement: Supplemental Information 1 [file peerj-cs-10-2607-s001.zip › code/attModel1/1.png]

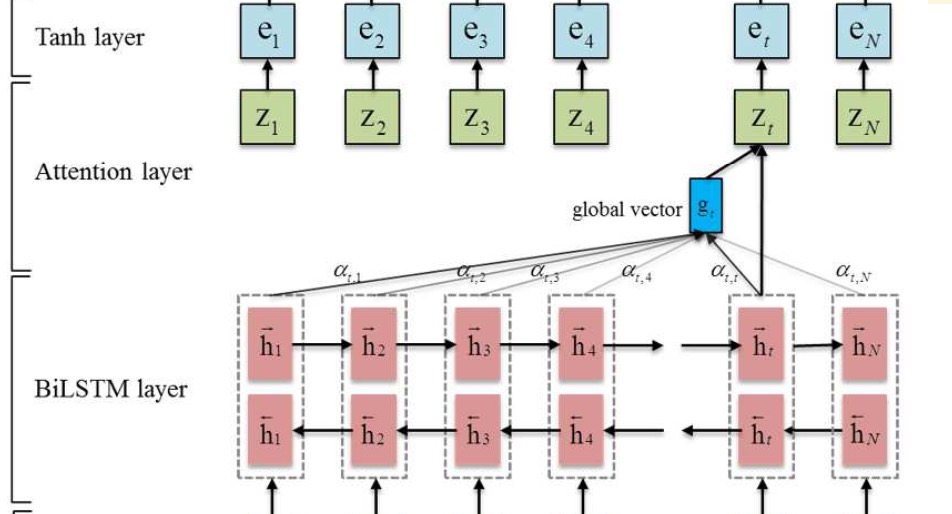

Supplement: Supplemental Information 1 [file peerj-cs-10-2607-s001.zip › code/attModel1-luoling/resAtt.png]

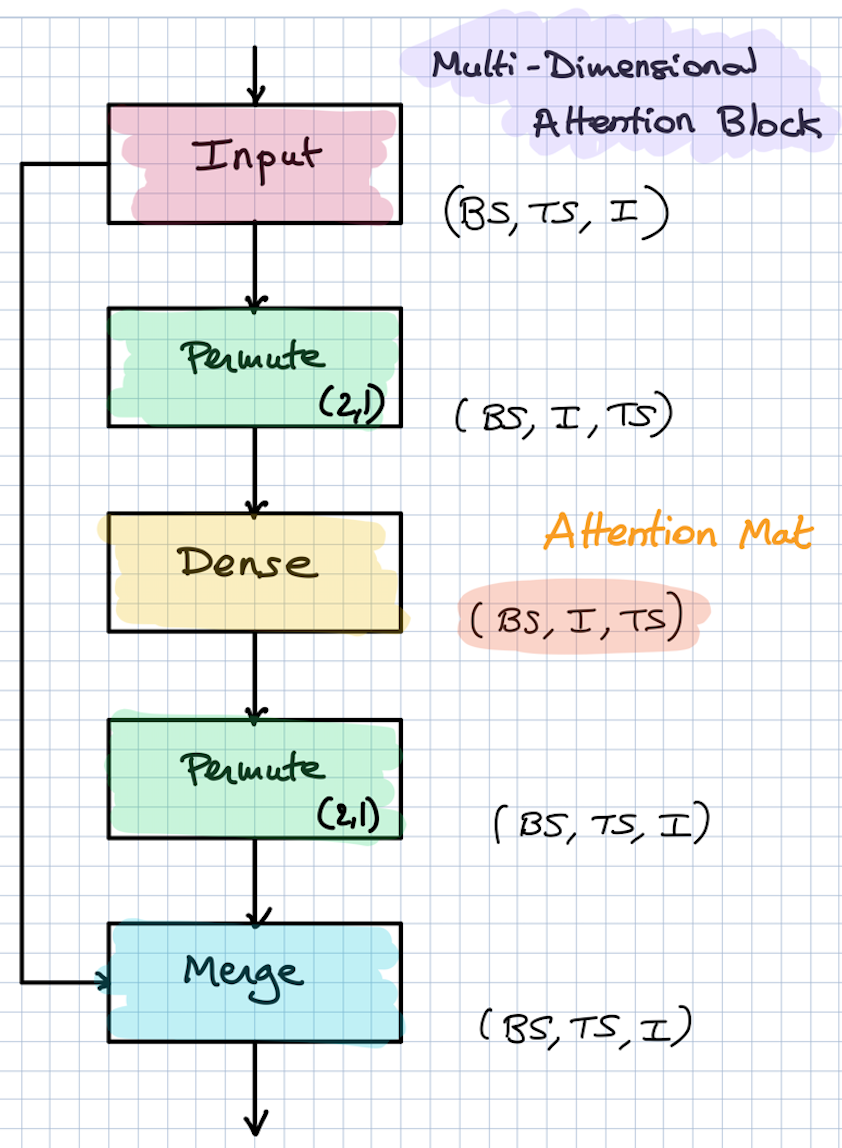

Supplement: Supplemental Information 1 [file peerj-cs-10-2607-s001.zip › code/attModel2/attention.png]

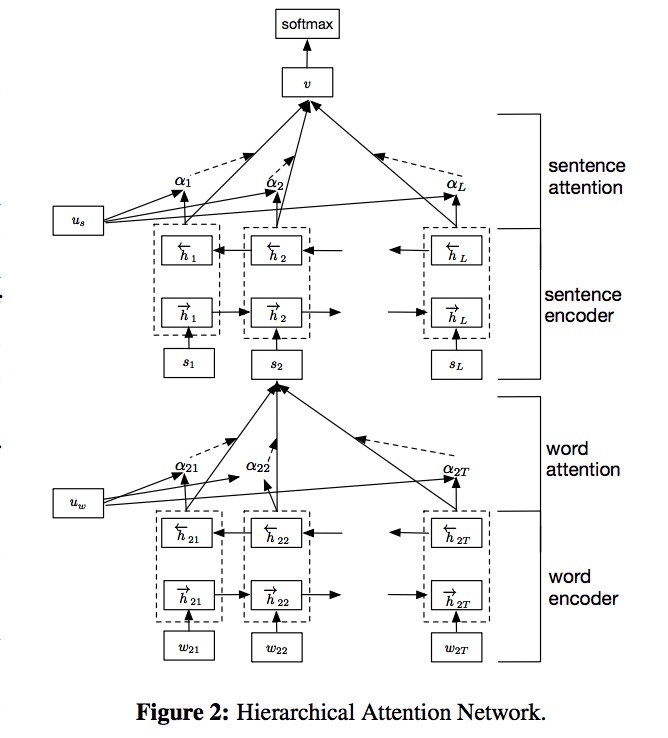

Supplement: Supplemental Information 1 [file peerj-cs-10-2607-s001.zip › code/hierarchical-attention_synthesio/1.png]

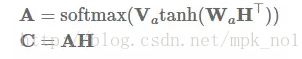

Supplement: Supplemental Information 1 [file peerj-cs-10-2607-s001.zip › code/self-attention/self.png]
